# Organising maternal and newborn care in high-income countries: a scoping review of organisational elements and their association with outcomes

**DOI:** 10.1136/bmjopen-2025-107624

**Published:** 2025-12-14

**Authors:** Jolanda Liebregts, Bahareh Goodarzi, Pim Valentijn, Soo Downe, Jan Jaap Erwich, George Burchell, Ronald Batenburg, Ank de Jonge, Corine J M Verhoeven, VOICE Study Group, K Burzynska

**Affiliations:** 1 Midwifery Science, Amsterdam UMC Locatie VUmc, Amsterdam, Netherlands; 2 Midwifery Academy Amsterdam Groningen, Inholland, Amsterdam, Netherlands; 3 Amsterdam Public Health, Quality of Care, Amsterdam UMC, locatie VUMC, Amsterdam, Netherlands; 4 The University of Groningen, University Medical Centre Groningen, Department of General Practice & Elderly Care Medicine, Groningen, Netherlands; 5 Essenburgh Research & Consultancy, Essenburgh Group, Harderwijk, Netherlands; 6 Research Group Nursing, Center of Expertise Healthy Ageing, Hanze University of Applied Sciences, Groningen, Netherlands; 7 ReaCH team, University of Central Lancashire, preston, UK; 8 Obstetrics and Gynaecology, University of Groningen, Groningen, Netherlands; 9 Medical Library, Vrije Universiteit Amsterdam, Amsterdam, Netherlands; 10 Netherlands Institute for Health Services Research, Utrecht, Netherlands; 11 Department of Sociology, Radboud University Nijmegen, Nijmegen, Netherlands; 12 Midwifery, School of Health Sciences, University of Nottingham, Nottingham, UK; 13 Amsterdam Reproduction and Development, Amsterdam, Netherlands; 14 Department of Obstetrics and Gynaecology, Maxima Medical Centre, Veldhoven, Netherlands

**Keywords:** Organisation of health services, OBSTETRICS, PERINATOLOGY, Patient-Centered Care

## Abstract

**Introduction:**

Countries face challenges in maternal and newborn care (MNC) regarding costs, workforce and sustainability. Organising integrated care is increasingly seen as a way to address these challenges. The evidence on the optimal organisation of integrated MNC in order to improve outcomes is limited.

**Objectives:**

(1) To study associations between organisational elements of integrated care and maternal and neonatal health outcomes, experiences of women and professionals, healthcare costs and care processes and (2) to examine how the different dimensions of integrated care, as defined by the Rainbow Model of Integrated Care, are reflected in the literature addressing these organisational elements.

**Results:**

We included 288 papers and identified 23 organisational elements, grouped into 6 categories: personal continuity of care; interventions to improve interdisciplinary collaboration and coordination; care by a midwife; alternative payment models (non-fee-for-service); place of birth outside the obstetric unit and woman-centred care. Personal continuity, care by a midwife and births outside obstetric units were most consistently associated with improved maternal and newborn outcomes, positive experiences for women and professionals and potential cost savings, particularly where well-coordinated multidisciplinary care was established. Positive professional experiences of collaboration depended on clear roles, mutual trust and respectful interdisciplinary behaviour. Evidence on collaboration interventions and alternative payment models was inconclusive. Most studies emphasised clinical and professional aspects rather than organisational integration, with implementation barriers linked to prevailing biomedical system orientations.

**Conclusions:**

Although the literature provides substantial evidence of organisational elements that contribute to improved outcomes, a significant gap remains in understanding how to overcome the barriers in sustainable implementation of these elements within healthcare systems. Interpreted through a systems and transition science lens, these findings suggest that strengthening integrated maternity care requires system-level changes aligning with WHO policy directions towards midwifery models of person-centred care.

**STRENGTHS AND LIMITATIONS OF THIS STUDY**

- The review was conducted by a multidisciplinary team, including client representatives, healthcare practitioners and researchers, which helped reduce bias during the review process.
- We used a systematic, AI-assisted screening tool (ASReview) to efficiently manage a large body of literature.
- The performance of the AI-assisted screening tool (ASReview) depends on the quality of the initial training data, which may have affected the efficiency of the screening process.
- The methodological design of a scoping review does not allow for evaluation of intervention effectiveness or certainty of evidence.
- The review focused on studies from high-income countries, limiting generalisability to low-income and middle-income settings.

## Introduction

 Around the world, maternal and newborn care (MNC) is facing increasing challenges, including a rise in medical interventions,^1 2^ a decline in workforce retention,^3^ rising healthcare costs^4 5^ and an increasing emphasis on the impact of a positive pregnancy and childbirth experience on the well-being of women.^6^ The latter leads to tensions between the flexibility and time that professionals and organisations need and have to meet women’s needs.^7^ The organisation of MNC should align with the overarching goals of the healthcare system: improving population health, enhancing the experiences of people, improving the well-being of professionals and limiting per capita costs.^8 9^ However, healthcare systems often prioritise disease detection and the treatment of complications over health promotion and prevention. This focus extends to MNC, where acute services are emphasised more than preventive and supportive care.^10^

Governments are trying to address these challenges by reforming healthcare organisations. Integrating care is seen as an enabling strategy.^11 12^ Integrated care emphasises coordinated efforts across various levels of the healthcare system and fosters collaboration within and between healthcare and social service organisations to improve continuity of care, enhance service efficiency, elevate patient experience and achieve better health outcomes.^12 13^ There is, however, no consensus in the international literature on how integrated care should be organised or how it can be achieved. This is due, for example, to different stakeholder perspectives and differences in healthcare organisations within and between countries.^11 14^

For this study, we adapted the definition of integrated care of Allana *et al*^15^ to the context of MNC; ‘network(s) of multiple professionals and organisations in maternal, newborn and social care that provide accessible, comprehensive and coordinated services to women planning a pregnancy, currently pregnant or within 6 weeks post partum’.^15^ Valentijn *et al* conceptualised integrated care in the Rainbow Model of Integrated Care (RMIC) in an attempt to capture its multidimensional nature.^16^ RMIC comprises four dimensions of integration: clinical integration (guidelines and protocols), professional integration (roles and responsibilities), organisational integration (aligning resources with healthcare organisation goals) and system integration (payment systems, legislation and policies). The model combines person-focused and population-based principles, showing, across dimensions of integrated care, various smaller ‘organisational elements’ (determinants), such as different models of risk selection, different models of antenatal, intrapartum and postnatal care and different payment models. Currently, little is known about the organisational elements of integrated MNC, their association with outcomes and how they are embedded within integrated care. Furthering this understanding is important to inform evidence-based integrated care initiatives within MNC. Therefore, in this scoping review, we explored the extent and type of evidence on: (a) organisational elements of integrated MNC and their associations with maternal and neonatal health outcomes, the experiences of women and healthcare professionals, healthcare costs and healthcare processes and (b) how the different dimensions of integrated care, as defined by the RMIC, are reflected in the literature on these organisational elements.

## Methods

This scoping review was conducted using the JBI methodology for scoping reviews^17^ and the PRISMA Extension for Scoping Reviews reporting guidelines.^18^ The objectives, inclusion criteria and the collaboration with a multiple stakeholder expert group were prespecified and described in our prospectively published study protocol (online supplemental file A).^19^ A preliminary search of MEDLINE, the Cochrane Database of Systematic Reviews and the JBI Evidence Synthesis identified no completed or ongoing systematic or scoping reviews on this topic. This study was conducted in accordance with the prospectively published protocol.^19^ We did not deviate from the planned methods, except that the data analysis process took longer than anticipated. To ensure that the review included the most recent and relevant studies, we performed an updated literature search and conducted an additional round of study selection.

### Search strategy

A systematic search was performed by an information specialist (GLB) in the following databases: PubMed, Scopus, PsycINFO and the Wiley/Cochrane Library, from 1 January 2012 to 24 August 2022, with an update on 30 October 2024. The following terms, including synonyms and closely related words, were used as index terms or free-text words; ‘integrated care’ combined with (synonyms of) ‘maternal and neonatal health’ or (synonyms of) ‘patient experience’ or (synonyms of) ‘healthcare professional’ or (synonyms of) ‘healthcare spending’ or (synonyms of) ‘care processes’. A full overview of the search terms per database can be found in online supplemental file B. No limitations on language were applied to the search. Publications from 2012 onwards were considered, as this was the year in which the WHO European Region’s Health 2020 policy was adopted. This policy prioritises health system strengthening and promotes people-centred health systems,^8^ marking a shift that has brought greater attention to integrated care. Grey literature and unpublished studies were explored through Google Scholar and key websites of interest (eg, WHO and government agencies). Potentially relevant references were also retrieved through the snowball method and through consultation with the expert group.

### Selection of publications, data extraction and analysis

We identified, collected and uploaded citations to EndNote V20,^4^ and duplicates were removed. We included publications in English and Dutch, describing the association between a specific element of organisation of MNC and one or more of the following outcomes: maternal and newborn health, women’s experiences, experiences of professionals, healthcare processes and/or healthcare costs. A specific element of organisation was considered an intervention or practice that can be implemented in MNC. No prior list of elements of organisation was used; the elements were identified in the publications using an inductive approach. Publications were excluded if the element of organisation was not specified or if they included multiple elements.

We conducted a systematic, two-stage screening process to evaluate the relevance of the publications identified in the search.^19^ In the first stage, two reviewers (JL and BG) screened the titles and abstracts of the publications independently to determine their eligibility. For this stage, we used an open-source, machine learning-assisted tool called ASReview, V.1.1.^20^ At the start of the screening process, both reviewers provided initial training data to the model by labelling a small number of publications as relevant or irrelevant, supplemented with a set of key articles on integrated MNC defined by the authors and an expert group. ASReview then used this information to prioritise the order in which the remaining records were presented for screening. The reviewers continued to screen titles and abstracts in the order suggested by the model, and their labelling decisions were fed to ASReview iteratively, allowing the algorithm to improve its predictions during the process. All inclusion and exclusion decisions were made by the reviewers and were cross-checked to ensure consistency. Since there was no protocol or recommendation available on when to stop screening, we defined the stopping rule based on the knowledge of the research team and factors such as time and available resources. Screening was stopped when either a maximum of 400 papers were identified as relevant or 50 papers were consecutively identified as irrelevant. We repeated this process, using the same criteria, for the search update of October 2024.

In the second stage, full-text screening was conducted using a staged calibration process. Two reviewers (JL and BG) independently assessed three consecutive sets of 25 full-texts. After each set, disagreements were resolved through discussion and, if needed, by consultation with a third reviewer (CJMV). As no further disagreements arose in the third set, JL completed screening of the remaining full texts, discussing any uncertainties with BG and CJMV.

JL, BG and CJMV independently extracted data from the included publications, using the data extraction tool in Excel developed by the research team.^19^ The following data were extracted: First author, year of publication, country of study origin, title, aim, study type, specific element of organisation and associations with one or more outcomes. To extract information on the organisational elements within the dimensions of integrated care, we employed a combination of inductive and deductive approaches. First, we coded the data inductively to identify the specific elements of organisation studied in the included studies. This was an iterative process conducted by the author team, during which codes referring to similar or overlapping organisational elements were discussed, refined and grouped into six broader categories of organisational elements that captured the main ways in which MNC was organised across the studies.

**Figure 1:**
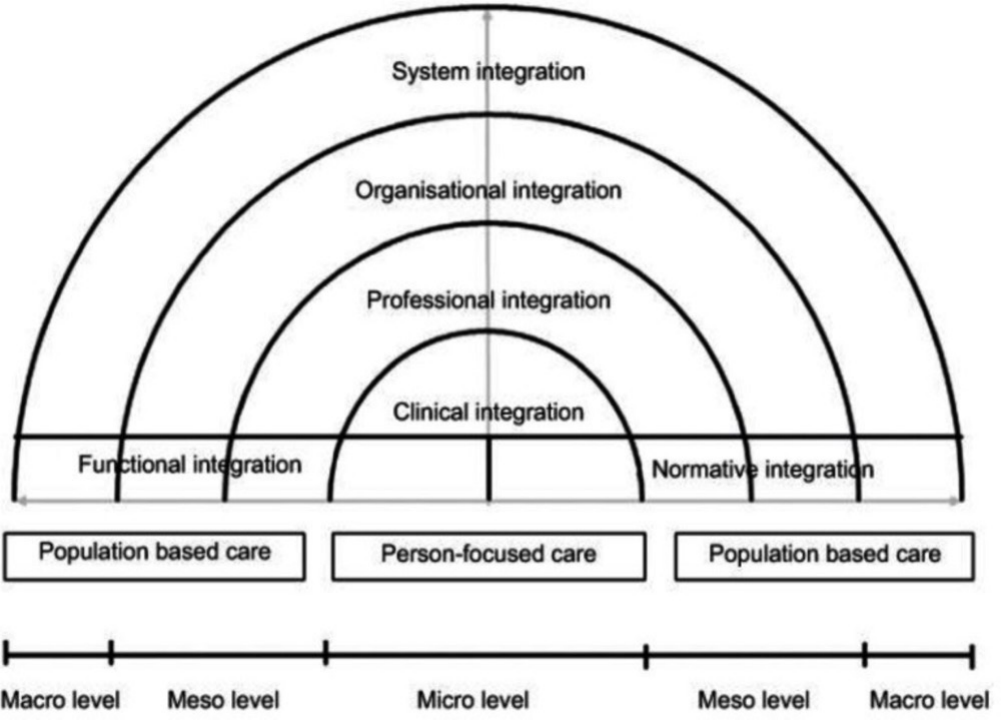
Rainbow Model of Integrated Care.^21^

In the next step, we applied a deductive approach informed by the theoretical framework of the RMIC (figure 1).^21^ Using the RMIC dimensions and their defined determinants, we mapped the information about each organisational element onto the corresponding dimensions of integrated care. Any disagreements between the reviewers were resolved through discussion or with a fourth reviewer (AdJ).

## Results

**Figure 2:**
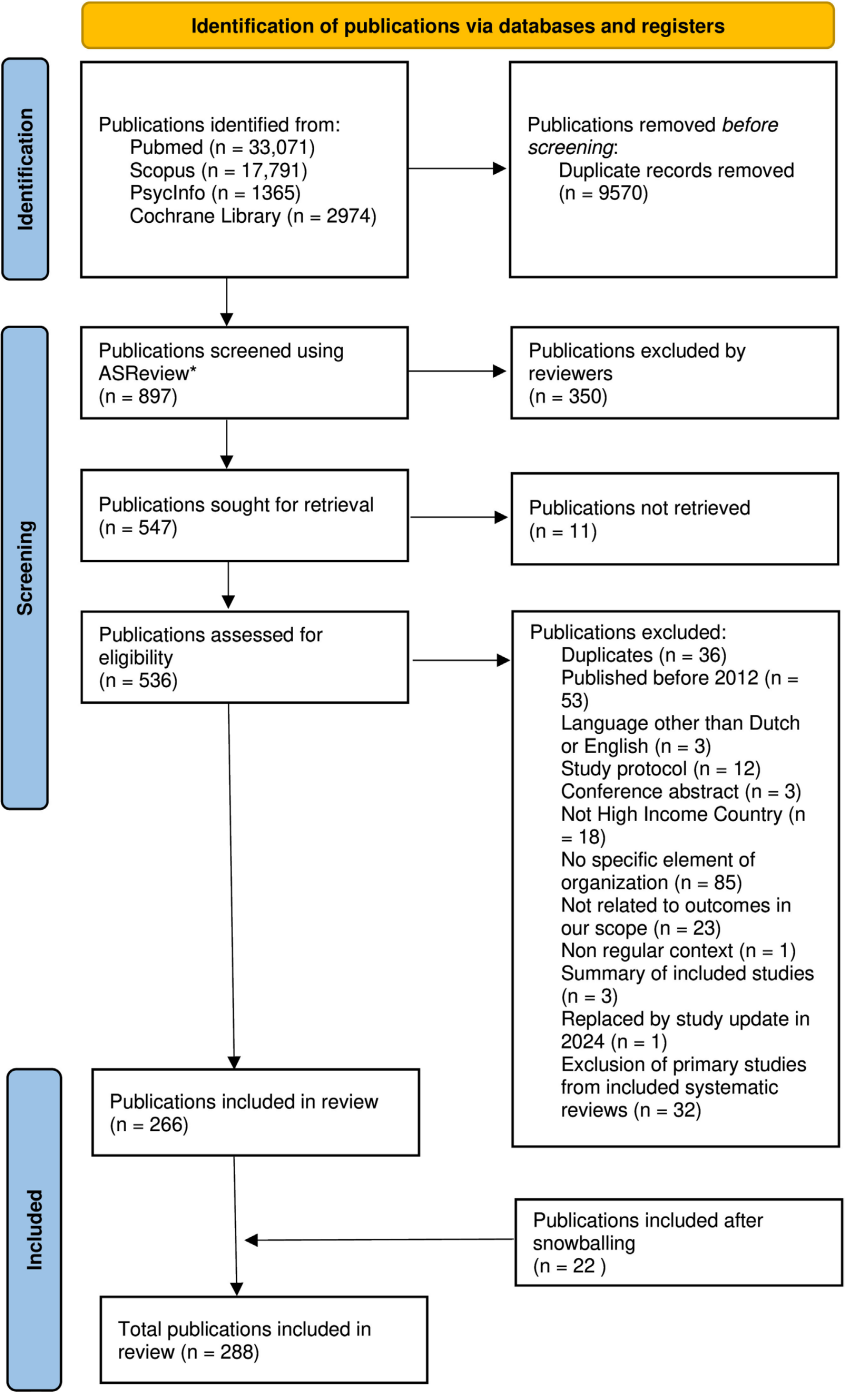
Preferred Reporting Items for Systematic reviews and Meta-Analyses extension for Scoping Reviews (PRISMA-ScR) flow diagram.^18^ *ASReview, version 1.1.^20^

The systematic search yielded 45 631 unique publications after duplicate entries were removed. Using ASReview, we screened a total of 897 titles and abstracts before reaching our stopping criterion. We marked 350 titles and abstracts as irrelevant, as they did not describe any element of organisation or outcomes within our scope. Consequently, 547 publications retrieved after the initial search and the update were left for full-text review. Of these, 11 publications could not be accessed, 227 publications did not meet our inclusion criteria, while 32 primary studies were part of included systematic reviews. Through snowballing, 22 publications were added, resulting in a total of 288 publications for analysis (figure 2).

### Characteristics of publications

Of the 288 publications included in this review, 270 (94%) were peer-reviewed and 18 (6%) were grey literature. The publications were published between 2012 and 2024 and originated from 23 high-income countries. Of the peer-reviewed studies, 37 were systematic reviews, 17 were randomised controlled trials (RCTs), 126 were quantitative (cohort, survey and retrospective studies), 55 were qualitative studies and 16 had a mixed-methods design. There were nine literature reviews, four scoping reviews and one realist review. The characteristics of the included publications can be found in online supplemental file C.

### Groups of elements of organisation and their association with outcomes

Using an inductive approach, we identified 23 specific elements of organisation and categorised them into 6 groups, based on their common characteristics. table 1 shows the characteristics of the publications per element of organisation of care and how these elements were clustered into the six groups: (1) personal continuity of care (n=118 publications); (2) interventions to foster interdisciplinary collaboration and coordination (n=19); (3) care by a midwife (n=73); (4) alternative payment model (other than fee-for-service) (n=8); (5) place of birth outside the obstetric unit (n=34 and (6) woman-centred care (n=36) (table 1).

**Table 1:** Characteristics of the included publications analysed by (groups of) organisational elements and research method

Included publications (n=288)			Research method					
Groups of elements of organisation of care	Elements of organisation of care	Total (%)	Systematic Review and Randomised controlled trial	Review (Scoping, realist, literature)	Quantitative (cohort–survey–retrospective–quasi experimental**)**	Qualitative	Mixed Methods	Grey literature
Personal continuity of care Elements of organisation to ensure personal continuity of care; women receive maternal and newborn care or support throughout pregnancy, labour and the postpartum period from one or a small team of care providers.	Continuous support by a doula	**2 (1)**	1	0	1	0	0	0
	Midwife-led continuity of care	**106 (37)**	14	7	48	24	10	3
	Private care by an obstetrician	**5 (2)**	0	0	4	0	1	0
	Private care by a midwife	**5 (2)**	0	0	4	1	0	0
Interventions to foster Interdisciplinary collaboration Papers explicitly describe the effect of interdisciplinary collaboration on outcomes.	Multidisciplinary consultations and care pathways	**6 (2)**	3	0	2	1	0	0
	Shared care (explicit shared interdisciplinary responsibility for the client)	**13 (5)**	3	0	5	1	1	3
Care by a midwife Elements of organisation where care is provided by a midwife as opposed to the standard, individual, hospital-based, doctor-led, fragmented care. Either antenatal, intrapartum or postpartum and/or for the whole care pathway. Personal continuity of care is not an aim or an explicit part of the practice described.	Continuous support during labour	**3 (1)**	1	0	1	1	0	0
	Group antenatal care	**18 (6**)	7	1	5	4	0	1
	Midwife-led antenatal care	**11 (4)**	3	1	4	3	0	0
	Midwife-led care - whole care path	**7 (2)**	1	0	4	1	0	1
	Midwife-led intrapartum care	**14 (5)**	1	2	9	1	0	1
	Midwife-led postnatal care	**16 (6)**	4	0	4	7	1	0
	Task shifting from medical specialists to community midwives	**4 (1)**	0	0	1	1	1	1
Alternative payment model (APM) (other than fee-for-service) Elements of organisation to adjust the remuneration of healthcare services.	Bundled Payment	**5 (2)**	0	1	4	0	0	0
	Allocated funding for autonomous midwives	**1 (0)**	0	0	0	1	0	0
	Salaried employment of obstetricians	**1 (0)**	0	0	1	0	0	0
	Pay for Performance	**1 (0)**	0	0	1	0	0	0
Place of birth—outside obstetric setting Elements of organisation describe the setting of giving birth at home or in a birth centre (alongside an obstetric unit or freestanding), in the care of a midwife.	Birth centre	**19 (7)**	1	1	11	4	0	2
	Home birth	**15 (5)**	3	0	9	0	1	2
Woman-centred care Elements of organisation that support individual women’s needs, promote shared decision-making, promote autonomy, respect individual circumstances and perspectives and enable working with women to strengthen their ability to care for themselves and their families.	Involvement of cultural brokers in the organisation of MNC care to ensure culturally informed community care	**5 (2)**	2	0	2	1	0	0
	Integrating women’s voices	**4 (1)**	0	0	1	1	0	2
	Shared decision-making	**4 (1)**	0	1	1	1	1	0
	Supportive care	**23 (8)**	10	0	9	2	0	2
Total per study design (%)		**288**	**54 (19)**	**14 (5)**	**131 (45)**	**55 (19)**	**16 (6)**	**18 (6)**

We described the associations with outcomes for the six groups of elements of organisation of MNC in table 2. Consistent with scoping review methodology, we reported on the outcomes that have been studied in relation to each organisational element. However, we did not assess the effectiveness or quality of the evidence.

**Table 2:** Associations between groups of organisational elements and five outcome domains

Outcomes Group of elements	Maternal and neonatal health	Experiences of women	Experiences of professionals	Healthcare processes	Healthcare spending
Personal continuity of care	(71)* 11 RCTs and systematic reviews showed that MLCC was associated with higher rates of spontaneous vaginal birth and lower rates of caesarean and instrumental delivery. Observational studies linked MLCC to reduced preterm birth, with some evidence of fewer episiotomies. Findings for neonatal mortality, maternal morbidity and breastfeeding were inconsistent, and no adverse effects were reported. Associations between MLCC and health outcomes were generally stronger when compared with standard hospital care than with standard midwife-led care. Private obstetric continuity was associated with more interventions, inductions and elective caesareans, but one study reported lower perinatal mortality.	(54) Publications on personal continuity of care showed more positive experiences during pregnancy, labour and in the postpartum period. MLCC was described to lead to more shared decision-making, more informed consent for medical interventions, culturally appropriate care and freedom of mobility during labour. Few publications showed positive experiences with private obstetric care; this may be related to clear patient communication, education and accessibility to resources.	(33) Personal continuity of care was described to be related to positive experiences for midwives through the development of meaningful relationships with women and obstetric colleagues. Publications showed that midwives having professional autonomy, working across the full scope of midwifery practice with an appropriate caseload, and with influence over workload and work–life balance, may experience lower rates of burnout.	(13) Publications showed that personal continuity of care was related to more time per consultations. In cases where there was continuity of care from a doula, women were more likely to attend childbirth-preparation classes. Personal continuity of care may lead to more space for health education.	(18) For personal continuity of care by midwives, publications showed a trend towards lower costs. For private obstetrician and private midwife care, restricted access was reported, due to a lack of local availability of private care and because women need to have private health insurance or self-fund.
Interventions to foster interdisciplinary collaboration and coordination	(13) Included publications reported that the use of risk selection tools to assign women to multidisciplinary care pathways was not consistently related to better outcomes for all women. One study found a reduction of perinatal adverse outcomes in nulliparous women. One study described a successful multidisciplinary audit and feedback that led to a lower c-section rate. Some evidence suggested that shared care may increase access to MNC for specific groups and reduce adverse perinatal outcomes due to attention to multiple risk factors within a multidisciplinary team.	(7) Studies reported negative experiences for women when communication was perceived as limited, fragmented and associated with conflicting advice. Positive experiences were described to be related to continuity of care and information, to comprehensive care-coordination, when care met the women’s needs, and when women perceived professionals’ interest in their emotional well-being, and when they experienced sufficient time to discuss their concerns.	(4) Studies showed more positive experiences when professionals perceived higher levels of interprofessional trust, role clarity and professional and considerate interdisciplinary behaviour. Publications showed negative experiences when the interdisciplinary collaboration between professionals of opposite philosophy of care (physiology as opposed to biomedical) was perceived not to be constructive.	(5) Multidisciplinary consultations and multidisciplinary care pathways were described to promote uniformity and structure in work routines. This may lead to a more proactive and preventive approach regarding medical and non-medical risk factors. Some publications showed that multidisciplinary consultations and multidisciplinary care pathways may lead to increased medicalisation.	(3) Few studies showed that multidisciplinary consultations and care pathways may reduce costs through reduced c-section rates. One study found that using a risk selection tool could be cost-effective.
Care by a midwife	(37) Publications showed that care by a midwife, compared with standard hospital-based obstetrician-led care, was associated with lower rates of interventions, caesarean section, preterm birth and postpartum haemorrhage and with higher rates of spontaneous vaginal birth and reduced newborn hospitalisation. No adverse maternal or neonatal outcomes were reported; some studies found no differences in caesarean, instrumental birth or epidural rates. GANC was associated with lower rates of preterm birth, low birth weight and NICU admission and reduced maternal depressive symptoms.	(37) Publications showed that women reported positive experiences related to a sense of control over labour pain and to their ability to make decisions. Positive or negative experiences with midwife-led postpartum care depended on the woman’s individual perspectives, circumstances and support networks. Publications reported positive experiences with GANC, related to women’s increased knowledge, confidence and ability to build a support network.	(14) Midwives reported higher satisfaction in models consistent with midwifery philosophy. In biomedical settings, ‘being with women’ was often unfamiliar, undervalued or constrained by workload. Several studies described frustration with active-management policies limiting autonomy and scope of practice. Publications on GANC reported positive experiences, including more effective use of time, improved continuity and professional role development in antenatal care.	(13) Referral rates from midwife-led to obstetrician-led care varied by local protocols and guidelines; only a small proportion of women were referred for urgent reasons. Appropriate risk selection by midwives was linked to early identification and timely management of complications, supported by efficient referral and transfer processes. Few publications associated community-based midwife-led postnatal care with earlier hospital discharge after birth.	(13) Publications showed that care by a midwife was associated with a cost reduction on national and individual level. Studies suggested that more analysis is needed to evaluate the cost-effectiveness regarding the lower rates of epidural analgesia, c-section and instrumental birth rates in midwife-led care. Few publications showed that GANC may be cost-effective.
APM (other than fee-for-service)	(8) Publications showed mixed and often limited evidence on associations with health outcomes. Most publications reported no significant changes in maternal or neonatal health outcomes. Where effects were observed, they were small, inconsistent or context-specific—such as slight shifts in c-section rates.	No data in the included publications.	No data in the included publications.	(2) Publications showed that APM, coupled with performance metrics, may improve processes of care and reduce some types of acute services. Unintended consequences were described; for example, emergency department visits as a substitute for additional physician visits.	(7) Evidence on association with costs was mixed and limited; publications showed that APMs were associated with modest cost reductions, mainly through lower prices rather than reduced service use. Emergency department visits as a substitute for additional physician visits may lead to cost-shifting and increased hospitalisations.
Place of birth—outside obstetric setting	(21) Most publications on births outside obstetric units reported lower intervention rates, lower severe maternal morbidity, higher spontaneous vaginal birth rates and greater perineal integrity for women starting care in birth centres compared with hospitals. Some studies indicated a trend towards lower caesarean rates, while others found no difference. No evidence of increased severe outcomes was reported for planned home births among low-risk women supported by trained midwives and effective referral systems.	(14) Publications reported more positive birth experiences among women with homebirth compared with birth centre, and birth centre compared with hospital settings. Transfer from planned homebirth to hospital was associated with feelings of uncertainty. A trusting relationship with the midwife was central to women’s sense of safety and well-being in hospital care. Women described self-efficacy and confidence, which may positively influence the transition to motherhood.	(3) Positive experiences were described for midwives providing comprehensive, personalised care, autonomously and independent from the obstetric ward. Midwives and obstetricians may experience challenges in bridging conflicting paradigms of safety and risk in case of transfer of care.	(7) Publications showed higher rates of transfer for nulliparous women than multiparous women.	(6) Cost savings per birth were described for women planning home birth compared with women planning a hospital birth with a midwife, and higher savings were described when compared with planned hospital birth with an obstetrician.
Woman-centred care	(21) No evidence was found to support the assumption that integrating women’s voices into organisational or quality cycles improves health outcomes. Supportive care and access to homelike birth environments, birthing pools and aids (eg, bean bags) were associated with reduced analgesia and intervention use and higher rates of spontaneous vaginal birth. Home visits by health visitors or public health nurses were not linked to improved outcomes, whereas visits based on relational continuity and shared cultural understanding were associated with better maternal and neonatal outcomes.	(15) Publications showed positive experiences for care in a homelike birth setting. Some evidence was found for more positive experiences with education during home visits by health visitors or nurses. In midwifery care, positive experiences of women were related to a more active role in collaborative decision-making; in private obstetric care, women were satisfied with a more passive role in decision-making.	(8) Publications showed that in a biomedical setting, professionals experienced barriers to support woman-centred and supportive care. Barriers were reported to be hierarchical decision-making and rationalisation of the routine use of clinical interventions. Described facilitators to support woman-centred care were; working in collaboration with cultural brokers, and collaborative working between midwives and obstetricians. Public health nurses had positive experiences with home visits as it helped in building a relationship with women.	No data in the included publications.	(2) Publications using economic modelling identified a decrease in the rise of healthcare costs if the public were fully engaged in their health.

* Number of publications with information on the outcome. Publications may have information on more than one outcome.

APM, alternative payment model; GANC, group antenatal care; MLCC, midwife led continuity of care; MNC, maternal and newborn care; NICU, neonatal intensive care unit; PTB, preterm birth; RCTs, randomised controlled trial.

Of all the publications included, 191 reported on the association between an element of organisation and maternal and neonatal health outcomes, 145 reported on the association with women’s experiences, 73 reported on associations with professionals’ experiences, 43 reported on the association with healthcare processes and 51 showed associations between elements of organisation and healthcare costs. Publications on a specific element of organisation of care often reported on associations with more than one outcome. In the literature, the elements of organisation under investigation were generally compared with the standard, hospital-based, doctor-led, fragmented care (219 publications). However, in 39 of the 106 studies focusing on personal continuity provided by midwives, this element was compared with standard care by midwives without personal continuity (known or unknown midwife, working rotational shifts in different services). We further present the evidence on the relationship between the groups of elements of organisation and their relationship with outcomes in table 2.

Included publications reported that personal continuity of care was associated with better health outcomes for mothers and children and positive experiences for mothers.^22^,^35^ Some studies also showed a trend towards lower costs.^2536^,^40^ When midwives could work autonomously and within their full scope of practice, with an appropriate caseload, midwife-led continuity of care was reported to be associated with positive experiences of midwives and may prevent burnout.^41^,^49^ Similarly, publications focussing on MNC provided by midwives rather than obstetricians (‘care by a midwife’), under the condition that acute services were timely available when needed, showed better maternal and neonatal health outcomes, mainly due to a reduction in interventions and an increase in spontaneous vaginal births.^50^,^57^ The positive association for women in midwife-led care was consistent in cohort studies and RCTs with similar groups of women receiving either care by midwives or standard hospital-based care by obstetricians.^2658^,^60^ Depending on the space given to the midwifery approach in the biomedical setting, publications showed that the experiences of women and midwives were more positive or more negative.^61^,^68^ In publications where midwives delivered MNC, costs were reduced due to lower salary costs and lower costs due to fewer interventions.^51 69 70^ In addition, positive associations with health outcomes and lower healthcare costs were seen in publications on ‘place of birth—outside the obstetric unit’.^6071^,^73^ Again, this was related to fewer interventions and higher rates of spontaneous vaginal birth. However, a prerequisite for birth outside the obstetric unit was an MNC system with well-trained midwives and a good referral and transport system for transfer to adequate medical and obstetric/neonatal care in the hospital setting.^71 72^ Women who gave birth outside the obstetric unit generally reported positive experiences, which were linked to personal care and perceived autonomy.^60 74 75^ Less consistent evidence was found regarding the relationship between ‘woman-centred care’ and health outcomes.^76^,^81^ Nevertheless, several publications indicated that positive experiences were associated with taking an active role in shared decision-making.^1082^,^84^ Evidence also shows that professionals perceived barriers to providing woman-centred care within a predominantly biomedical system, characterised by efficient, protocol-driven and risk-averse practices, high intervention rates and a hierarchical structure favouring medical specialists over other healthcare professionals and clients.^7783 85^,^87^ Collaboration with cultural brokers to provide culturally informed care was found to facilitate woman-centred care and enhance professionals’ experiences.^88^,^91^

Included publications showed that ‘interventions to foster interdisciplinary collaboration and coordination’, such as multidisciplinary care pathways for women in vulnerable situations, may ensure better access to the right care provider and more uniform care provision.^92^,^95^ Little and inconsistent evidence was found for associations with health outcomes or care costs.^9293 95^,^97^ Women’s experiences were shown to be dependent on the level of consistency in communication and information between healthcare professionals.^98 99^ For professionals, positive experiences of collaboration depended on role clarity, trust and considerate interdisciplinary behaviour.^96 100^ Also, for ‘alternative to fee-for-service payment models’, we found mixed and limited evidence on associations with improved health outcomes or lower costs.^5101^,^105^

22 publications addressed limited access to the elements of organisation as a key constraint on their potential impact on outcomes.^2327 106^,^110^ Authors identified multiple barriers to accessing elements of organisation, particularly personal continuity of care, care by a midwife, place of birth outside the obstetric unit and woman-centred care.^111^,^113^ The information about and coordination of these services often lacked a uniform and effective approach, leading to limited access for women.^87114^,^117^ Availability was frequently cited as a limiting factor, with services often concentrated in urban centres and limited or absent in rural and remote areas.^2740 118^,^120^ Workforce shortages, particularly of midwives and community-based providers, further constrained service provision.^110 121 122^ Financial barriers—including lack of private health insurance and out-of-pocket costs for midwifery care in some jurisdictions —disproportionately affected low-income women, limiting their access to both public and private care options.^31 107 123^ Institutional factors such as short hospital stay, limited appointment time and centralised models of care reduced opportunities for relational and individualised support.^87 124 125^ Additionally, systemic issues such as language barriers, low health literacy and challenges navigating healthcare systems particularly affected migrant and socially disadvantaged populations.^27126^,^128^ Authors emphasised the need for locally accessible, culturally safe, and adequately resourced models of care that enable early engagement and sustained support throughout the perinatal period.^26 110 129 130^

### Groups of elements of organisation: information on the dimensions of the RMIC

Having defined the elements of organisation and their associations with outcomes, we examined what had been written about the organisation of integrated care in the included publications through the RMIC lens. In all the publications included, we identified information relating to (some of the) elements of organisation across the RMIC model’s six dimensions of integrated care (table 3).^21^

**Table 3:** Division of the publications by the groups of elements of organisation and the different dimensions of the Rainbow Model of Integrated Care (RMIC)

Group of elements RMIC’s dimension	Personal continuity of care (118)	Interventions to foster interdisciplinary collaboration and coordination (19)	Care by a midwife (73)	Alternative payment model (APM) (other than fee-for-service) (8)	Place of birth–outside the obstetric unit (34)	Woman-centred care (36)
Clinical integration*	(118)^†^ Personal continuity of care involved one or a small team of professionals providing and coordinating care throughout pregnancy, birth and the postpartum period. Organisation varied across studies, including caseload or group midwifery models and private obstetric care. Key components included trusting relationships and informed consent. Client involvement was described through shared decision-making. No publications addressed case management or individual multidisciplinary care plans.	(5)Two publications addressed client involvement: one through a mother council of women from diverse backgrounds and another through implementation of shared decision-making within a multidisciplinary care pathway. Three publications focused on multidisciplinary care pathways and consultations aimed at improving continuity and coordination of care, described mainly in terms of informational and management continuity.	(73) Midwives provided some or all antenatal, intrapartum and postpartum care, with or without continuity. It can be organised in group antenatal care in the antenatal period, mostly by community midwives. Midwives tend to promote the physiology of pregnancy and childbirth through personalised care and support for intrapartum mobilisation using birthing pool and aids. Two publications addressed the care coordination by midwives and one paper mentioned client involvement through shared decision-making.	(0)	(34) Option for low-risk women to give birth at home or in a birth centre (alongside an obstetric unit or freestanding), in the care of a midwife.	(7) Some publications suggested that client involvement may lead to empowerment of women in shared decision-making, to promoting autonomy and to access to clear and timely information about care options and interventions. It was also suggested that clients can be involved in different levels of organisation, one study showed that client involvement often did not go beyond the level of consultation (Arnstein participation ladder).
Professional integration	(5) Effective, respectful interdisciplinary collaboration and clear role definitions were essential for providing personal continuity of care. Midwives’ scope of practice varied across countries; legal ability to continue care alongside medical colleagues for women with pre-existing conditions or complications enhanced continuity. A shared professional vision, intentional team-building and leadership promoting respect and cooperation were identified as key facilitators.	(19) Findings showed the defined roles, competencies and activities of the different professionals in the multidisciplinary collaboration. Shared care and multidisciplinary care pathways, consultations and interdisciplinary training and education were described to foster interdisciplinary collaboration and consultation, as well as to promote access to care and quality of care for people in vulnerable situations.	(3) Collaboration between midwives and professionals in a biomedical setting was often described as limited through tensions over role boundaries and power dynamics.	(0)	(2) Findings showed the need for interdisciplinary risk selection, referral guidelines and multidisciplinary consultations about referrals in between facilities including emergency and non-emergency transport.	(8) Client involvement in quality improvement required cross-organisational systems, time, expertise and institutional support. Multidisciplinary, team-based approaches enhanced engagement. Studies emphasised respect for women’s autonomy, though information on intervention risks and benefits was often insufficient. Clinical decision-making remained predominantly biomedical, favouring routine over personalised care. Community-governed, interprofessional models, especially those co-developed with Indigenous communities, facilitated culturally informed care, with midwives central in linking community and hospital services.
Organisational Integration	(0)	(1) One study described hospital collaboration as a learning network but provided no details on contracting, agreements or governance.	(0)	(0)	(0)	(0)
System integration	(0)	(0)	(0)	(1) Systemwide programmes may yield smaller effects than voluntary ones.	(0)	(0)
Functional integration	(34) Adequate staffing, institutional flexibility and strong leadership were identified as essential to support personal continuity of care. Allowing midwives to move in and out of caseload models supported sustainability and work–life balance. Legislative and organisational support was required to enable midwives to work to their full scope of practice, including in complex care. Robust information-sharing systems were addressed to be essential for informational continuity. Consensus on continuity definitions, standardised perinatal data and integration with community-based care was needed for system-level planning.	(5) Findings highlighted the need for robust information-sharing systems to ensure informational and managerial continuity across multidisciplinary care pathways. Communication tools were required to support clients and professionals during transfers between care providers and between health and social services. One publication emphasised ongoing monitoring, evaluation and adaptation of shared care through network governance. Training in teamwork and involvement of implementation experts was suggested to strengthen interdisciplinary collaboration and coordination.	(17) Care by a midwife was facilitated by adequate resource allocation, dedicated infrastructure such as freestanding units and secure shared medical records. Improved evaluation of birth outcomes was needed to inform scale-up. Reported barriers included staff shortages, rigid scheduling and institutional time constraints limiting midwifery practice. Digital tools were seen to enhance information flow. Group care models required investment in training, facilities for group sessions and revised scheduling systems.	(7) Findings highlighted inconsistent terminology and ambiguous definitions that hinder understanding of APMs. Publications emphasised the need for clear definitions and predefined goals developed collaboratively before implementation, including agreed criteria for assessing success. Linking shared savings to quality metrics was discussed as a potential strategy to support achievement of quality targets and effective implementation of APMs.	(5) Findings indicated the need for licensure and accreditation of birth centres, supported by one electronic patient record to improve referral quality. Effective risk selection and referral systems were required. Multidisciplinary audits of severe maternal morbidity were suggested to enhance care quality and collaboration. Reported barriers to community birth settings included limited midwifery workforce growth and inconsistent or unsustainable reimbursement for midwifery and birth centre services.	(8) Findings highlighted the need for greater coherence and collaboration among professionals to strengthen client participation at national, regional and local levels. Policies promoting shared decision-making and a quality framework to guide workforce development and resource allocation were recommended. A formal cross-organisational quality improvement system driven by women’s input was needed, supported by enhanced data collection and expertise. Effective communication during care transfers and coordinated, integrated services was essential to ensure informational and management continuity.
Normative integration	(34) Findings indicated that normative integration of personal continuity of care depended on alignment between midwifery and biomedical philosophies. Lack of shared values, trust and interdisciplinary respect hindered implementation and scale-up. Midwifery philosophy emphasised autonomy, relational continuity and holistic, woman-centred care, whereas the biomedical approach prioritised risk management, standardisation and clinical control. This tension impeded collaboration and system coherence. One publication suggested a continuum balancing both approaches to foster shared values and interprofessional understanding.	(3) Authors emphasised the need for mutual respect, trust and motivation among professionals to implement multidisciplinary care pathways. Establishing shared goals and ambitions across professions and organisations was seen to promote collaboration. Roles were described as complementary and should enable responsiveness to changing needs of women, families and care contexts. Integrated practice combining midwifery and obstetrics was viewed as beneficial, with respect for both philosophies and recognition of their continuum supporting effective collaboration.	(15) Findings indicated that normative integration of midwife-led care was challenged by tensions between midwifery philosophy and biomedical models prioritising efficiency, control and risk management. Midwives’ relational, woman-centred approaches were often undervalued within institutional cultures, weakening professional identity and interprofessional collaboration. Alignment of care values, mutual respect and inclusion of midwives in planning and reform processes were identified as essential to embed care by midwives within standard, hospital-based obstetric systems.	(5) Findings indicated that fee-for-service funding may create competition for births, restrict midwives’ scope and hospital access, and inadequately compensate obstetricians as midwifery volumes increase. The siloed funding of midwives, obstetricians and hospitals, combined with the absence of a coordinated health workforce plan, limited opportunities for midwives to address system gaps and reduced their influence in decisions affecting their level of integration.	(5) Findings supported implementation of birth options outside obstetric units through integration of midwife-led units and home births. Adopting a midwifery philosophy was seen to promote respectful, individualised care. Birth settings were associated with differing professional values and approaches to labour management, while women choosing birth centres or home births often held distinct philosophies compared with those opting for obstetric unit births.	(15) Findings highlighted normative barriers to integrating woman-centred care, which often conflicted with biomedical, risk-based models reinforced by hierarchy, professional boundaries and institutionalised intervention norms. Midwifery’s individualised and relational philosophy was constrained by systemic pressures, limiting autonomy and reinforcing medical dominance. Additional barriers included limited stakeholder participation, poor responsiveness to women’s experiences and lack of continuity. System-level change towards collaborative, culturally competent and woman-led care models was recommended to support woman-centred care integration.

* Dimensions of the RMIC with their determinants^21^: Clinical integration: The extent to which care services for clients are coordinated across various professional, institutional and sectorial boundaries within a system. Determinants: Case management, continuity, individual multidisciplinary care plan and client participation. Professional integration: Partnerships between professionals. Related to the professionals’ degree of collective responsibility to provide a continuous, comprehensive and coordinated continuum of care. Determinants: Interprofessional education, shared vision between professionals, multidisciplinary guidelines and protocols, and interprofessional governance. Organisational integration: Refers to interorganisational relationships, including shared mechanisms to provide comprehensive services to a population. Determinants: Performance-management, learning organisations, complaints procedure, interest management. System integration: Refers to a tailored combination of (in)formal rules and policies between care providers and external stakeholders for the benefit of people and populations. Determinants: Stakeholder management, environmental climate, available resources, good governance. Functional integration: Refers to key support functions and activities around the primary process of service delivery, to coordinate and support accountability and decision-making between organisations and professionals to add overall value to the system. Determinants: Information management, service management, regular feedback on performance indicators. Normative integration: Refers to the development and maintenance of a common frame of reference between organisations, professional groups and individuals. Determinants: Experienced trust, visionary leadership, quality feature of the informal collaboration, reliable behaviour

† Number of publications in the group of elements with information on the dimension of integration. Publications may have information on more than one dimension. The total may be more than the total number of publications per group of elements.

We found most information on what had been done specifically in practice regarding the implementation of the elements of organisation at the dimension of clinical and professional integration. Authors addressed the importance of care coordination by one or a small team of professionals in the provision of personalised continuity of care.^4244 131^,^133^ Also, the importance of role clarity between professionals, respecting each other’s roles, the need for interprofessional communication skills and fostering collaboration was addressed.^49 118 121 134^ We found that across the groups of elements where midwives play a role, authors addressed a need for their professional autonomy.^29 48 49 135 136^

We found limited information on elements of integrated care at the organisational or system dimension of integration. None of the included studies addressed aspects at the organisational level, such as contracts, alliances or mergers, to implement the element of organisation. One study reported a collaboration between hospitals to implement an audit and education programme aimed at improving the quality of care. However, no details were shared in the publication on how this collaboration was organised in terms of governance.^137^ Also, there was no information on what was done specifically at the dimension of system integration, eg, addressing the role of the economic, political or social environment in which the element of organisation was implemented. The influence of policy on the organisation of care was described in three studies on alternative payment systems. These studies were conducted in the context of local policy reforms towards bundled payments,^102 103 138^ and one review referred to the ‘Better Birth’ policy reform in the UK.^139^

At the level of functional and normative integration, we found a large amount of information on what would be needed to make the element reach its potential. The authors did not specifically describe what exactly was done on these dimensions to bring about the effect of the element of organisation. Authors across all groups of elements of organisation emphasised the necessity of robust systems for information sharing and standardised perinatal data collection, as well as a cross-organisational quality improvement system based on such data, including client self-reported outcomes and experiences.^10 27 34 44 85 98 104 105 140^ On the dimension of normative integration, a recurring theme was the standard, biomedical model of care, which limited the implementation of personalised continuity of care, care by a midwife, place of birth outside the obstetric unit and woman-centred care.^8384 106 141^,^143^ Biomedical settings, defined by efficient, protocol-driven, risk-averse practices, high intervention rates and a hierarchy of medical specialists over other healthcare professionals and clients, were described as hindering midwives’ autonomy and decision-making power.^87^ Decision-making was described to prioritise routine interventions over personalised care, and centralised care in obstetric units was described to enforce ‘institutional time’, promoting routine clinical interventions and prioritising efficiency over individualised care.^6877 83 136 139 141 144^,^149^ As a normative enabler for personal continuity of care, authors recommended adopting a more physiological philosophy of MNC within societies.^1061 150^,^152^

Authors argued that this philosophical tension between the biomedical and midwifery philosophies of care hindered collaboration and system-wide support for midwifery care.^135 146 153^ The findings suggested that a continuum that respects both approaches may be essential in facilitating shared values, mutual respect, trust, interprofessional collaboration and care coordination.^10 100 154^

## Discussion

We conducted this review to enhance the understanding of the association between organisational elements of integrated MNC and outcomes. We identified six groups of elements of organisation of care: ‘personal continuity of care’, ‘interventions to foster interdisciplinary collaboration and coordination’, ‘care by a midwife’, ‘alternative (to fee-for-service) payment model’, ‘place of birth outside the obstetric unit’ and ‘woman-centred care’.

We found substantial evidence showing an association between personal continuity of care, care by a midwife and birth outside the obstetric unit, and improved maternal health outcomes, as well as more positive experiences for both women and midwives. These findings could not be explained by a difference in risk factors, as studies included groups of women that were similar at the onset of care and that remained in these groups for the analyses, regardless of their care processes. Improved outcomes were related to lower intervention rates and higher rates of spontaneous vaginal births. Evidence showed that greater autonomy was related to enhanced experiences for both midwives and women. Conversely, systems based on routine obstetrician-led, hospital-based or fragmented care were shown to be associated with structural barriers to woman-centred care, leading to more negative experiences. Notably, a significant number of publications on the needs of women and professionals in fostering more positive experiences and achieving better outcomes were excluded from our review as they did not address specific organisational elements to achieve these effects. Evidence for the effectiveness of interdisciplinary collaboration and alternative payment models on health outcomes or costs was limited and inconsistent.

Using the RMIC framework, the evidence we found was primarily focused on clinical and professional integration, with limited attention given to the RMC elements of organisational and system-level integration. Functional integration showed a clear need for robust systems to facilitate information exchange, standardised perinatal data collection and cross-organisational quality improvement based on clinical data and patient-reported outcomes and experiences. Consistent barriers were found on the normative dimension in terms of limitations in access and the sustainable implementation of personalised continuity of care, care by a midwife and birth outside the obstetric unit. These barriers were rooted in the dominant biomedical model of MNC in high-income countries, characterised by efficient, protocol-driven, risk-averse practices, high intervention rates and a hierarchy of medical specialists over other healthcare professionals and clients. Within this model, both clients and professionals who want to work in a more woman-centred way reported having limited autonomy, time and control in accessing or providing alternatives to standard care.

The emphasis on information on organisation at the client-professional level is also seen in the wider literature on integrated care. The scope of studies on organisation of care in relation to outcomes often focuses on the interventions at the client-provider level, where the precise implementation and active mechanisms are often not described and thus form a ‘black box’. The organisational dimension of integrated care is often under-reported. This results in an incomplete picture of what exactly is organised to explain the relationships found with outcomes.^155^,^157^ This may provide a one-sided picture of interventions that increase access to care or show positive effects on outcomes. Unforeseen effects on interests, finances or hierarchy on organisational or system level dimensions can then be such that further implementation is hindered. Integrated care scholars therefore address the importance of examining the complexity of organisation across multiple dimensions of integrated care and its nonlinearity in the context of the effectiveness of integrated care.^158 159^

In our review, we found many studies that showed associations between care by a midwife, personal continuity of care (mainly by midwives) and place of birth outside the medical setting (where midwives provide care) and positive outcomes. Recently, the WHO has called for a transition to midwifery models of care in which midwives play a pivotal role in close collaboration with other maternity care professionals.^160^ These models have been associated with improved maternal and neonatal outcomes, increased satisfaction and more efficient use of resources, reflecting the core components observed in our analysis.^127 160^ The WHO describes midwifery models of care as ‘models of care in which midwives work autonomously within their scope of practice, they collaborate as members of interdisciplinary teams, within networks of care, to ensure continuous, integrated and collaborative care that is respectful and cost-effective’.^160^

Midwifery models of care add physiological, psychological, social and cultural factors to the biomedical approach to MNC. In the broader literature on models of care, this is called the biopsychosocial approach to health (BPS); it conceptualises health and illness as the result of dynamic interactions between biological, psychological and social factors. It recognises the role of individual behaviour, mental health, environmental context and social relationships in shaping health outcomes. Care based on this model emphasises patient-centredness, shared decision-making and holistic assessment.^161^,^163^ Within this approach, continuity of care is also studied in hospital settings, where increased continuity of care by doctors has been associated with lower mortality rates.^164 165^ In general primary care, it has been associated with decreased utilisation of health services, including rehospitalisation and emergency visits, as well as lower healthcare costs.^164^,^169^

A requirement for the safety of birth outside the obstetric unit is that this care is well integrated into the healthcare system.^170^ The large prospective Birthplace in England cohort study published in 2011 showed that, compared with planned hospital birth, low-risk multiparous women who planned to give birth in either alongside or freestanding birth centres, or at home, had better outcomes, with no differences for neonates. This was also the case for primiparous women, except for those planning home birth, who had slightly higher adverse neonatal outcomes.^171^ The more recent systematic reviews by Hutton *et al* and Reitsema *et al* showed that, in jurisdictions where home birth is well-integrated into the healthcare system, planned home births are associated with comparably low rates of serious adverse neonatal outcomes and a lower incidence of intrapartum interventions compared with planned hospital births.^71 170^

Our review includes publications that addressed the biomedical settings as a barrier to implementing alternatives to standard care. This finding is shared by Zarbiv *et al*.^172^ They found barriers to the implementation of midwife-led care, which they attributed to systemic hierarchical power dynamics, limited midwife autonomy, workforce shortages and inadequate policy support.^172^ Although hierarchy can contribute to the efficient organisation of care, Essex *et al*^173^ argue that the high costs of inequalities in status, power and the resulting negative effects on staff retention, outweigh its benefits.^173^ Simmelink *et al*^174^ revealed that structural, financial and organisational barriers, such as the incompatibility of current maternity care systems with small team models, as well as personal factors and professional tensions, were key obstacles to implementing continuity of care in midwifery practice.^174^ They suggested that client advocacy could play an important role in the adaptation and sustainability of organising personal continuity of care by midwives.

The biomedical model was frequently reported to act as a barrier for implementation of organisational improvements in MNC within larger, relatively stable healthcare systems. When viewed through a systems and transition science lens, such stability can be understood as part of existing ‘regimes’, that tend to resist change, even when alternative organisational approaches are well-described in the evidence base.^175^ This resistance arises because transition involves not only developing new ways of working but also letting go of deeply rooted routines, structures and power dynamics. Interpreted in this way, the implementation of organisational improvements in MNC represents a system-level transition from the existing regime rather than a simple adoption of best practices—an understanding that provides valuable context for interpreting the findings of this review.

### Strengths and limitations

This review was conducted by a multidisciplinary team, including client representatives, healthcare practitioners and researchers, which helped reduce bias during the review process. We used a systematic, AI-assisted screening tool (ASReview) to efficiently manage a large body of literature, although its performance depends on the quality of the initial training data and may have affected screening efficiency. The scoping review design does not allow for evaluation of intervention effectiveness or certainty of evidence. Finally, the review focused on studies from high-income countries, which may limit the generalisability of the findings to other contexts.

## Conclusions

This scoping review mapped the existing evidence on organisational elements of integrated MNC and identified six overarching groups of organisational elements. Across the included studies, personal continuity of care, care provided by a midwife and birth outside the obstetric unit were frequently reported to be associated with positive maternal and neonatal outcomes, improved experiences among women and professionals and indications of lower costs. Implementation and wider adoption of these organisational elements were frequently reported to be hampered by the prevailing biomedical—risk averse, disease focused, protocolised—orientation of health systems in many high-income countries.

When interpreted through the lens of systems and transition science, these findings suggest that strengthening integrated MNC involves more than the adoption of discrete best practices. It represents a system-level transition that requires shifts in organisational routines, professional relationships and underlying paradigms of care. The findings align with the direction outlined in recent WHO policy, which calls for a transition towards midwifery models of care—person-centred care in which midwives play a pivotal role in close collaboration with other maternity care professionals. Further research is needed to explore the mechanisms and contextual conditions that can support and sustain such a transition within health systems.

## Supplementary material

10.1136/bmjopen-2025-107624 online supplemental file 1

10.1136/bmjopen-2025-107624 online supplemental file 2

10.1136/bmjopen-2025-107624 online supplemental file 3

## Data Availability

Data are available on reasonable request.
