# Supplementary material for: Organising maternal and newborn care in high-income countries: a scoping review of organisational elements and their association with outcomes
Source: BMJ Open. 2025 Dec 14;15(12):e107624. doi: 10.1136/bmjopen-2025-107624 (PMC12706211; doi:10.1136/bmjopen-2025-107624)
Supplement: online supplemental file 2 [file bmjopen-15-12-s002.pdf]

Supplementary file A

Search terms per database

PubMed

Search	Query	Results
#6	from 2012 - 2025	33,008
#5	#4 NOT ("Developing Countries"[Mesh] OR "developing countr*"[tiab] OR "developing nation*"[tiab] OR "developing population*"[tiab] OR "developing econom*"[tiab] OR "undeveloped countr*"[tiab] OR "undeveloped nation*"[tiab] OR "undeveloped economy"[tiab] OR "undeveloped economies"[tiab] OR "least developed countr*"[tiab] OR "least developed nation*"[tiab] OR "least developed economy"[tiab] OR "least developed economies"[tiab] OR "less-developed countr*"[tiab] OR "less-developed nation*"[tiab] OR "less-developed population"[tiab] OR "less-developed populations"[tiab] OR "less-developed econom*"[tiab] OR "lesser developed countr*"[tiab] OR "lesser developed nation*"[tiab] OR "lesser developed population"[tiab] OR "lesser developed populations"[tiab] OR "lesser developed economy"[tiab] OR "lesser developed economies"[tiab] OR "under-developed countr*"[tiab] OR "under-developed nation*"[tiab] OR "underdeveloped countr*"[tiab] OR "underdeveloped nation*"[tiab] OR "underdeveloped population*"[tiab] OR "underdeveloped econom*"[tiab] OR "low income countr*"[tiab] OR "middle income countr*"[tiab] OR "low income nation*"[tiab] OR "middle income nation*"[tiab] OR "low income population*"[tiab] OR "middle income population*"[tiab] OR "low income econom*"[tiab] OR "middle income econom*"[tiab] OR "lower income countr*"[tiab] OR "lower income nation*"[tiab] OR "lower income population*"[tiab] OR "lower income economy"[tiab] OR "lower income economies"[tiab] OR "resource limited"[tiab] OR "low resource countr*"[tiab] OR "lower resource countr*"[tiab] OR "low resource nation*"[tiab] OR "low resource population*"[tiab] OR "low resource economy"[tiab] OR "low resource economies"[tiab] OR "underserved countr*"[tiab] OR "underserved nation*"[tiab] OR "underserved population*"[tiab] OR "underserved economy"[tiab] OR "underserved economies"[tiab] OR "under-served country"[tiab] OR "under-served countries"[tiab] OR "under-served nation"[tiab] OR "under-served nations"[tiab] OR "under-served population"[tiab] OR "under-served populations"[tiab] OR "underserved economy"[tiab] OR "underserved economies"[tiab] OR "deprived countr*"[tiab] OR "deprived nation"[tiab] OR "deprived nations"[tiab] OR "deprived population*"[tiab] OR "deprived economy"[tiab] OR "deprived economies"[tiab] OR "poor countr*"[tiab] OR "poor nation*"[tiab] OR "poor population*"[tiab] OR "poor econom*"[tiab] OR "poorer countr*"[tiab] OR "poorer nation*"[tiab] OR "poorer population*"[tiab] OR	63,561

Search	Query	Results
	"poorer econom*"[tiab] OR "lmic"[tiab] OR "lmics"[tiab] OR "lami"[tiab] OR "transitional countr*"[tiab] OR "transitional nation"[tiab] OR "transitional nations"[tiab] OR "transitional econom*"[tiab] OR "transition countr*"[tiab] OR "transition nation*"[tiab] OR "transition econom*"[tiab] OR "low resource setting*"[tiab] OR "lower resource setting*"[tiab] OR "middle resource setting*"[tiab] OR "Third World*"[tiab])	
#4	#1 AND #2 AND #3	68,136
#3	Search: **preconception care"[MeSH Terms] OR "prenatal care"[MeSH Terms] OR "Pregnancy"[MeSH] OR "pregnant women"[MeSH Terms] OR "perinatal care"[MeSH Terms] OR "postnatal care"[MeSH Terms] OR "obstetrics"[MeSH Terms] OR "Midwifery"[Mesh] OR "prenatal care"[tiab] OR Antenatal[tiab] OR Preconception*[tiab] OR Maternal*[tiab] OR Maternity[tiab] OR postnat*[tiab] OR Postpartum[tiab] OR perinat*[tiab] OR Prenatal*[tiab] OR "birth care*"[tiab] OR Pregnan*[tiab] OR Midwive*[tiab] OR midwifery[tiab] OR obstetri*[tiab] OR Neonat*[tiab] OR Newborn*[tiab] OR "New born*"[tiab] OR "birth attendant*"[tiab] OR gynaecolog*[tiab] OR gynecolog*[tiab] OR "natal care*"[tiab]**	1,877,060
#2	"maternal health"[MeSH Terms] OR "Pregnancy Outcome"[MeSH] OR "Pregnancy Complications"[Mesh] OR "infant health"[MeSH Terms] OR "Infant Mortality"[Mesh] OR "Morbidity"[Mesh] OR "Mortality"[Mesh] OR "Obstetric Labor Complications"[Mesh] OR "maternal health"[tiab] OR "infant health"[tiab] OR "neonatal health"[tiab] OR "neonate health"[tiab] OR "complications"[sb] OR "complicat*"[tiab] OR "adverse effects"[sb] OR "adverse effect*"[tiab] OR "adverse affect*"[tiab] OR "adversely affect*"[tiab] OR "side effect*"[tiab] OR "side affect*"[tiab] OR "adverse event*"[tiab] OR morbid*[tiab] OR death*[tiab] OR mortalit*[tiab] OR injur*[tiab] OR "adverse outcome*"[tiab] OR "Patient Satisfaction"[MeSH] OR ((Patient*[tiab] OR client*[tiab] OR "Women"[Mesh] OR Woman*[tiab] OR Women*[tiab]) AND (experience*[tiab] OR satisfaction*[tiab])) OR (("health personnel"[MeSH Terms] OR "Nurse Midwives"[Mesh] OR "Obstetrics"[Mesh] OR midwife*[tiab] OR midwiv*[tiab] OR Nurse*[tiab] OR "birth attendant*"[tiab] OR Obstetrician*[tiab] OR gynaecolog*[tiab] OR gynecolog*[tiab] Caregiver*[tiab] OR "care giver*"[tiab] OR Provider*[tiab] OR "health personnel"[tiab] OR "personnel health"[tiab]) AND (experience*[tiab] OR satisfaction*)) OR spending*[tiab] OR expenditure*[tiab] OR cost*[tiab] OR "Referral and Consultation"[Mesh] OR "patient transfer"[MeSH Terms] OR "Medicalization"[Mesh] OR Referral*[tiab] OR Intervention*[tiab] OR Transfer*[tiab] OR medicalization*[tiab] OR medicalisation*[tiab]	8,807,445

Search	Query	Results
#1	"Intersectoral Collaboration"[Mesh] OR "Models, Organizational"[Mesh] OR "Delivery of Health Care, Integrated"[Mesh] OR "Organization and Administration"[Mesh] OR "integrated care*"[tiab] OR "integrated maternity care*"[tiab] OR "integration of care*"[tiab] OR "Integration of maternity care"[tiab] OR "transmural care"[tiab] OR "continuity of care"[tiab] OR "continuum of care"[tiab] OR "model of care*"[tiab] OR "models of care*"[tiab] OR "care model*"[tiab] OR "care system*"[tiab] OR "risk selection*"[tiab] OR "Shared care"[tiab] OR "Joint care"[tiab] OR "Collaborative care"[tiab] OR Interprofessional*[tiab] OR "Inter professional*"[tiab] OR Collaboration*[tiab] OR Interdisciplinair*[tiab] OR Multidisciplinair[tiab] OR Cooperat*[tiab] OR "co oporat*"[tiab]	2,098,968

Scopus

Search	Query	Results
#7	#6 AND 2012-2025	18,031
#6	#5 NOT (conference paper OR conference review)	27,032
#5	#4 NOT TITLE-ABS-KEY ("developing countr*" OR "developing nation*" OR "developing population*" OR "developing econom*" OR "undeveloped countr*" OR "undeveloped nation*" OR "undeveloped economy" OR "undeveloped economies" OR "least developed countr*" OR "least developed nation*" OR "least developed economy" OR "least developed economies" OR "less-developed countr*" OR "less-developed nation*" OR "less-developed population" OR "less-developed populations" OR "less-developed econom*" OR "lesser developed countr*" OR "lesser developed nation*" OR "lesser developed population" OR "lesser developed populations" OR "lesser developed economy" OR "lesser developed economies" OR "under-developed countr*" OR "under-developed nation*" OR "underdeveloped countr*" OR "underdeveloped nation*" OR "underdeveloped population*" OR "underdeveloped econom*" OR "low income countr*" OR "middle income countr*" OR "low income nation*" OR "middle income nation*" OR "low income population*" OR "middle income population*" OR "low income econom*" OR "middle income econom*" OR "lower income countr*" OR "lower income nation*" OR "lower income population*" OR "lower income economy" OR "lower income economies" OR "resource limited" OR "low resource countr*" OR "lower resource countr*" OR "low resource nation*" OR "low resource population*" OR "low resource economy" OR "low resource economies" OR "underserved countr*" OR "underserved nation*" OR "underserved population*" OR "underserved economy" OR "underserved economies" OR "under-served country" OR "under-served countries" OR "under-served nation" OR "under-served nations" OR "under-served population" OR "under-served populations" OR "underserved economy" OR "underserved economies" OR "deprived countr*" OR "deprived nation" OR "deprived nations" OR "deprived population*" OR "deprived economy" OR "deprived economies" OR "poor countr*" OR "poor nation*" OR "poor population*" OR "poor econom*" OR "poorer countr*" OR "poorer nation*" OR "poorer population*" OR "poorer econom*" OR "lmic" OR "lmics" OR "lami" OR "transitional countr*" OR "transitional nation" OR "transitional nations" OR "transitional econom*" OR "transition countr*" OR "transition nation*" OR "transition econom*" OR "low resource setting*" OR "lower resource setting*" OR "middle resource setting*" OR "Third World*")	27,648
#4	#1 AND #2 AND #3	31,761,911

Search	Query	Results
#3	TITLE-ABS-KEY ("prenatal care" OR Antenatal OR Preconception* OR Maternal* OR Maternity OR postnat* OR Postpartum OR perinat* OR Prenatal* OR "birth care*" OR Pregnan* OR Midwive* OR midwifery OR obstetri* OR Neonat* OR Newborn* OR "New born*" OR "birth attendant*" OR gynaecolog* OR gynecolog* OR "natal care*")	31,141
#2	TITLE-ABS-KEY ("maternal health" OR "infant health" OR "neonatal health" OR "neonate health" OR "complicat*" OR "adverse effect*" OR "adverse affect*" OR "adversely affect*" OR "side effect*" OR "side affect*" OR "adverse event*" OR morbid* OR death* OR mortalit* OR injur* OR "adverse outcome*" OR ((Patient* OR client* OR Woman* OR Women*) AND (experience* OR satisfaction*)) OR ((midwife* OR midwiv* OR Nurse* OR "birth attendant*" OR Obstetrician* OR gynaecolog* OR gynecolog* Caregiver* OR "care giver*" OR Provider* OR "health personnel" OR "personnel health") AND (experience* OR satisfaction*)) OR spending* OR expenditure* OR cost* OR Referral* OR Intervention* OR Transfer* OR medicalization* OR medicalisation*)	17,741,999
#1	TITLE-ABS-KEY (“integrated care*" OR "integrated maternity care*" OR "integration of care*" OR "Integration of maternity care" OR "transmural care" OR "continuity of care" OR "continuum of care" OR "model of care*" OR "models of care*" OR "care model*" OR "care system*" OR "risk selection*" OR "Shared care" OR "Joint care" OR "Collaborative care" OR Interprofessional* OR "Inter professional*" OR Collaboration* OR Interdisciplinair* OR Multidisciplinair OR Cooperat* OR "co oporat*")	1,556,210

PsycINFO

Search	Query	Results
#5	S4 NOT (DE "Developing Countries" OR TI("developing countr*" OR "developing nation*" OR "developing population*" OR "developing econom*" OR "undeveloped countr*" OR "undeveloped nation*" OR "undeveloped economy" OR "undeveloped economies" OR "least developed countr*" OR "least developed nation*" OR "least developed economy" OR "least developed economies" OR "less-developed countr*" OR "less-developed nation*" OR "less-developed population" OR "less-developed populations" OR "less-developed econom*" OR "lesser developed countr*" OR "lesser developed nation*" OR "lesser developed population" OR "lesser developed populations" OR "lesser developed economy" OR "lesser developed economies" OR "under-developed countr*" OR "under-developed nation*" OR "underdeveloped countr*" OR "underdeveloped nation*" OR "underdeveloped population*" OR "underdeveloped econom*" OR "low income countr*" OR "middle income countr*" OR "low income nation*" OR "middle income nation*" OR "low income population*" OR "middle income population*" OR "low income econom*" OR "middle income econom*" OR "lower income countr*" OR "lower income nation*" OR "lower income population*" OR "lower income economy" OR "lower income economies" OR "resource limited" OR "low resource countr*" OR "lower resource countr*" OR "low resource nation*" OR "low resource population*" OR "low resource economy" OR "low resource economies" OR "underserved countr*" OR "underserved nation*" OR "underserved population*" OR "underserved economy" OR "underserved economies" OR "under-served country" OR "under-served countries" OR "under-served nation" OR "under-served nations" OR "under-served population" OR "under-served populations" OR "underserved economy" OR "underserved economies" OR "deprived countr*" OR "deprived nation" OR "deprived nations" OR "deprived population*" OR "deprived economy" OR "deprived economies" OR "poor countr*" OR "poor nation*" OR "poor population*" OR "poor econom*" OR "poorer countr*" OR "poorer nation*" OR "poorer population*" OR "poorer econom*" OR "lmic" OR "lmics" OR "lami" OR "transitional countr*" OR "transitional nation" OR "transitional nations" OR "transitional econom*" OR "transition countr*" OR "transition nation*" OR "transition econom*" OR "low resource setting*" OR "lower resource setting*" OR "middle resource setting*" OR "Third World*") OR AB("developing countr*" OR "developing nation*" OR "developing population*" OR "developing econom*" OR "undeveloped countr*" OR "undeveloped nation*" OR "undeveloped economy" OR "undeveloped economies" OR "least developed countr*" OR "least developed nation*" OR "least developed economy" OR "least developed economies" OR "less-developed countr*" OR "less-developed nation*" OR "less-developed population" OR "less-developed populations" OR "less-developed econom*" OR "lesser developed countr*" OR "lesser developed nation*" OR "lesser developed population" OR "lesser developed populations" OR "lesser developed economy" OR "lesser developed economies" OR "under-developed countr*" OR "under-developed nation*" OR "underdeveloped countr*" OR "underdeveloped nation*" OR "underdeveloped population*" OR "underdeveloped econom*" OR "low income countr*" OR "middle income countr*" OR "low income nation*" OR "middle income nation*" OR "low income population*" OR "middle income population*" OR "low income econom*" OR "middle income	1358

Search	Query	Results
	econom*" OR "lower income countr*" OR "lower income nation*" OR "lower income population*" OR "lower income economy" OR "lower income economies" OR "resource limited" OR "low resource countr*" OR "lower resource countr*" OR "low resource nation*" OR "low resource population*" OR "low resource economy" OR "low resource economies" OR "underserved countr*" OR "underserved nation*" OR "underserved population*" OR "underserved economy" OR "underserved economies" OR "under-served country" OR "under-served countries" OR "under-served nation" OR "under-served nations" OR "under-served population" OR "under-served populations" OR "underserved economy" OR "underserved economies" OR "deprived countr*" OR "deprived nation" OR "deprived nations" OR "deprived population*" OR "deprived economy" OR "deprived economies" OR "poor countr*" OR "poor nation*" OR "poor population*" OR "poor econom*" OR "poorer countr*" OR "poorer nation*" OR "poorer population*" OR "poorer econom*" OR "lmic" OR "lmics" OR "lami" OR "transitional countr*" OR "transitional nation" OR "transitional nations" OR "transitional econom*" OR "transition countr*" OR "transition nation*" OR "transition econom*" OR "low resource setting*" OR "lower resource setting*" OR "middle resource setting*" OR "Third World*") OR KW("developing countr*" OR "developing nation*" OR "developing population*" OR "developing econom*" OR "undeveloped countr*" OR "undeveloped nation*" OR "undeveloped economy" OR "undeveloped economies" OR "least developed countr*" OR "least developed nation*" OR "least developed economy" OR "least developed economies" OR "less-developed countr*" OR "less-developed nation*" OR "less-developed population" OR "less-developed populations" OR "less-developed econom*" OR "lesser developed countr*" OR "lesser developed nation*" OR "lesser developed population" OR "lesser developed populations" OR "lesser developed economy" OR "lesser developed economies" OR "under-developed countr*" OR "under-developed nation*" OR "underdeveloped countr*" OR "underdeveloped nation*" OR "underdeveloped population*" OR "underdeveloped econom*" OR "low income countr*" OR "middle income countr*" OR "low income nation*" OR "middle income nation*" OR "low income population*" OR "middle income population*" OR "low income econom*" OR "middle income econom*" OR "lower income countr*" OR "lower income nation*" OR "lower income population*" OR "lower income economy" OR "lower income economies" OR "resource limited" OR "low resource countr*" OR "lower resource countr*" OR "low resource nation*" OR "low resource population*" OR "low resource economy" OR "low resource economies" OR "underserved countr*" OR "underserved nation*" OR "underserved population*" OR "underserved economy" OR "underserved economies" OR "under-served country" OR "under-served countries" OR "under-served nation" OR "under-served nations" OR "under-served population" OR "under-served populations" OR "underserved economy" OR "underserved economies" OR "deprived countr*" OR "deprived nation" OR "deprived nations" OR "deprived population*" OR "deprived economy" OR "deprived economies" OR "poor countr*" OR "poor nation*" OR "poor population*" OR "poor econom*" OR "poorer countr*" OR "poorer nation*" OR "poorer population*" OR "poorer econom*" OR "lmic" OR "lmics" OR "lami" OR "transitional countr*" OR "transitional nation" OR "transitional nations" OR "transitional econom*" OR "transition countr*" OR "transition nation*" OR "transition	

Search	Query	Results
	econom*" OR "low resource setting*" OR "lower resource setting*" OR "middle resource setting*" OR "Third World*")) <br>2012 - 2025	
#4	#1 AND #2 AND #3	1949
#3	DE "Prenatal Care" OR DE "Childbirth Training" OR DE "Pregnancy" OR DE "Adolescent Pregnancy" OR OR DE "Primipara" OR DE "Perinatal Period" OR DE "Postnatal Period" OR DE "Obstetrics" OR DE "Caesarean Birth" OR DE "Midwifery" OR TI("prenatal care" OR Antenatal OR Preconception* OR Maternal* OR Maternity OR postnat* OR Postpartum OR perinat* OR Prenatal* OR "birth care*" OR Pregnan* OR Midwive* OR midwifery OR obstetri* OR Neonat* OR Newborn* OR "New born*" OR "birth attendant*" OR gynaecolog* OR gynecolog* OR "natal care*") OR AB("prenatal care" OR Antenatal OR Preconception* OR Maternal* OR Maternity OR postnat* OR Postpartum OR perinat* OR Prenatal* OR "birth care*" OR Pregnan* OR Midwive* OR midwifery OR obstetri* OR Neonat* OR Newborn* OR "New born*" OR "birth attendant*" OR gynaecolog* OR gynecolog* OR "natal care*") OR KW("prenatal care" OR Antenatal OR Preconception* OR Maternal* OR Maternity OR postnat* OR Postpartum OR perinat* OR Prenatal* OR "birth care*" OR Pregnan* OR Midwive* OR midwifery OR obstetri* OR Neonat* OR Newborn* OR "New born*" OR "birth attendant*" OR gynaecolog* OR gynecolog* OR "natal care*")	184,409
#2	DE "Pregnancy Outcomes" OR DE "Birth" OR DE "Induced Abortion" OR DE "Obstetrical Complications" OR DE "Spontaneous Abortion" OR DE "Morbidity" OR DE "Premorbidity" OR DE "Mortality Rate" OR DE "Mortality Risk" OR DE "Client Satisfaction" OR DE "Client Transfer" OR TI("maternal health" OR "infant health" OR "neonatal health" OR "neonate health" OR "complicat*" OR OR "adverse effect*" OR "adverse affect*" OR "adversely affect*" OR "side effect*" OR "side affect*" OR "adverse event*" OR morbid* OR death* OR mortalit* OR injur* OR "adverse outcome*" OR spending* OR expenditure* OR cost* OR Referral* OR Intervention* OR Transfer* OR medicalization* OR medicalisation*) OR AB("maternal health" OR "infant health" OR "neonatal health" OR "neonate health" OR "complicat*" OR OR "adverse effect*" OR "adverse affect*" OR "adversely affect*" OR "side effect*" OR "side affect*" OR "adverse event*" OR morbid* OR death* OR mortalit* OR injur* OR "adverse outcome*" OR spending* OR expenditure* OR cost* OR Referral* OR Intervention* OR Transfer* OR medicalization* OR medicalisation*) OR KW("maternal health" OR "infant health" OR "neonatal health" OR "neonate health" OR "complicat*" OR OR "adverse effect*" OR "adverse affect*" OR "adversely affect*" OR "side effect*" OR "side affect*" OR "adverse event*" OR morbid* OR death* OR mortalit* OR injur* OR "adverse outcome*" OR spending* OR expenditure* OR cost* OR Referral* OR Intervention* OR Transfer* OR medicalization* OR medicalisation*) OR	1,088,955

Search	Query	Results
	TI((Patient* OR client* OR OR Woman* OR Women* OR midwife* OR midwiv* OR Nurse* OR "birth attendant*" OR Obstetrician* OR gynaecolog* OR gynecolog* Caregiver* OR "care giver*" OR Provider* OR "health personnel" OR "personnel health") N3 (experience* OR satisfaction*)) OR AB((Patient* OR client* OR OR Woman* OR Women* OR midwife* OR midwiv* OR Nurse* OR "birth attendant*" OR Obstetrician* OR gynaecolog* OR gynecolog* Caregiver* OR "care giver*" OR Provider* OR "health personnel" OR "personnel health") N3 (experience* OR satisfaction*)) OR KW((Patient* OR client* OR OR Woman* OR Women* OR midwife* OR midwiv* OR Nurse* OR "birth attendant*" OR Obstetrician* OR gynaecolog* OR gynecolog* Caregiver* OR "care giver*" OR Provider* OR "health personnel" OR "personnel health") N3 (experience* OR satisfaction*))	
#1	DE "Integrated Services" OR TI("integrated care*" OR "integrated maternity care*" OR "integration of care*" OR "Integration of maternity care" OR "transmural care" OR "continuity of care" OR "continuum of care" OR "model of care*" OR "models of care*" OR "care model*" OR "care system*" OR "risk selection*" OR "Shared care" OR "Joint care" OR "Collaborative care" OR Interprofessional* OR "Inter professional*" OR Collaboration* OR Interdisciplinair* OR Multidisciplinair OR Cooperat* OR "co oporat*") OR AB("integrated care*" OR "integrated maternity care*" OR "integration of care*" OR "Integration of maternity care" OR "transmural care" OR "continuity of care" OR "continuum of care" OR "model of care*" OR "models of care*" OR "care model*" OR "care system*" OR "risk selection*" OR "Shared care" OR "Joint care" OR "Collaborative care" OR Interprofessional* OR "Inter professional*" OR Collaboration* OR Interdisciplinair* OR Multidisciplinair OR Cooperat* OR "co oporat*") OR KW("integrated care*" OR "integrated maternity care*" OR "integration of care*" OR "Integration of maternity care" OR "transmural care" OR "continuity of care" OR "continuum of care" OR "model of care*" OR "models of care*" OR "care model*" OR "care system*" OR "risk selection*" OR "Shared care" OR "Joint care" OR "Collaborative care" OR Interprofessional* OR "Inter professional*" OR Collaboration* OR Interdisciplinair* OR Multidisciplinair OR Cooperat* OR "co oporat*")	147,630

Cochrane

Search	Query	Results
#6	#5 AND 2012-2025	2539
#5	#4 NOT (developing NEXT countr* OR developing NEXT nation* OR developing NEXT population* OR developing NEXT econom* OR undeveloped NEXT countr* OR undeveloped NEXT nation* OR undeveloped NEXT economy OR undeveloped NEXT economies OR least NEXT developed NEXT countr* OR least NEXT developed NEXT nation* OR least NEXT developed NEXT economy OR least NEXT developed NEXT economies OR less-developed NEXT countr* OR less-developed NEXT nation* OR less-developed NEXT population OR less-developed NEXT populations OR less-developed NEXT econom* OR lesser NEXT developed NEXT countr* OR lesser NEXT developed NEXT nation* OR lesser NEXT developed NEXT population OR lesser NEXT developed NEXT populations OR lesser NEXT developed NEXT economy OR lesser NEXT developed NEXT economies OR under-developed NEXT countr* OR under-developed NEXT nation* OR underdeveloped NEXT countr* OR underdeveloped NEXT nation* OR underdeveloped NEXT population* OR underdeveloped NEXT econom* OR low NEXT income NEXT countr* OR middle NEXT income NEXT countr* OR low NEXT income NEXT nation* OR middle NEXT income NEXT nation* OR low NEXT income NEXT population* OR middle NEXT income NEXT population* OR low NEXT income NEXT econom* OR middle NEXT income NEXT econom* OR lower NEXT income NEXT countr* OR lower NEXT income NEXT nation* OR lower NEXT income NEXT population* OR lower NEXT income NEXT economy OR lower NEXT income NEXT economies OR resource NEXT limited OR low NEXT resource NEXT countr* OR lower NEXT resource NEXT countr* OR low NEXT resource NEXT nation* OR low NEXT resource NEXT population* OR low NEXT resource NEXT economy OR low NEXT resource NEXT economies OR underserved NEXT countr* OR underserved NEXT nation* OR underserved NEXT population* OR underserved NEXT economy OR underserved NEXT economies OR under-served NEXT country OR under-served NEXT countries OR under-served NEXT nation OR under-served NEXT nations OR under-served NEXT population OR under-served NEXT populations OR underserved NEXT economy OR underserved NEXT economies OR deprived NEXT countr* OR deprived NEXT nation OR deprived NEXT nations OR deprived NEXT population* OR deprived NEXT economy OR deprived NEXT economies OR poor NEXT countr* OR poor NEXT nation* OR poor NEXT population* OR poor NEXT econom* OR poorer NEXT countr* OR poorer NEXT nation* OR poorer NEXT population* OR poorer NEXT econom* OR lmic OR lmics OR lami OR transitional NEXT countr* OR transitional NEXT nation OR transitional NEXT nations OR transitional NEXT econom* OR transition NEXT countr* OR transition NEXT nation* OR transition NEXT econom* OR low NEXT resource NEXT setting* OR lower NEXT resource NEXT setting* OR middle NEXT resource NEXT setting* OR Third NEXT World*):ti,ab,kw	2794

Search	Query	Results
#4	#1 AND #2 AND #3	2,977
#3	(prenatal NEXT care OR Antenatal OR Preconception* OR Maternal* OR Maternity OR postnat* OR Postpartum OR perinat* OR Prenatal* OR birth NEXT care* OR Pregnan* OR Midwive* OR midwifery OR obstetri* OR Neonat* OR Newborn* OR New NEXT born* OR birth NEXT attendant* OR gynaecolog* OR gynecolog* OR natal NEXT care*):ti,ab,kw	152,665
#2	(maternal NEXT health OR infant NEXT health OR neonatal NEXT health OR neonate NEXT health OR complicat* OR adverse NEXT effect* OR adverse NEXT affect* OR adversely NEXT affect* OR side NEXT effect* OR side NEXT affect* OR adverse NEXT event* OR morbid* OR death* OR mortalit* OR injur* OR adverse NEXT outcome* OR ((Patient* OR client* OR Woman* OR Women*) AND (experience* OR satisfaction*)) OR ((midwife* OR midwiv* OR Nurse* OR birth NEXT attendant* OR Obstetrician* OR gynaecolog* OR gynecolog* NEXT Caregiver* OR care NEXT giver* OR Provider* OR health NEXT personnel OR personnel NEXT health) AND (experience* OR satisfaction*)) OR spending* OR expenditure* OR cost* OR Referral* OR Intervention* OR Transfer* OR medicalization* OR medicalisation*):ti,ab,kw	1,287,737
#1	(integrated NEXT care* OR integrated NEXT maternity NEXT care* OR integration NEXT of NEXT care* OR Integration NEXT of NEXT maternity NEXT care OR transmural NEXT care OR continuity NEXT of NEXT care OR continuum NEXT of NEXT care OR model NEXT of NEXT care* OR models NEXT of NEXT care* OR care NEXT model* OR care NEXT system* OR risk NEXT selection* OR Shared NEXT care OR Joint NEXT care OR Collaborative NEXT care OR Interprofessional* OR Inter NEXT professional* OR Collaboration* OR Interdisciplinair* OR Multidisciplinair OR Cooperat* OR co NEXT oporat*):ti,ab,kw	42,504
