# Supplementary material for: Organising maternal and newborn care in high-income countries: a scoping review of organisational elements and their association with outcomes
Source: BMJ Open. 2025 Dec 14;15(12):e107624. doi: 10.1136/bmjopen-2025-107624 (PMC12706211; doi:10.1136/bmjopen-2025-107624)
Supplement: online supplemental file 3 [file bmjopen-15-12-s003.pdf]

First author	Year	Country	Title	Element of Organisation	Group of elements of organisation	DOI
**Adams**	2017	Australia	Perinatal mortality disparities between public care and private obstetrician-led care: a propensity score analysis	Private care obstetrician	Personal continuity of care	10.1111/1471-0528.14903
**Adelson**	2023	Australia	Evaluation of a regional midwifery caseload model of care integrated across five birthing sites in South Australia: Women’s experiences and birth outcomes	MLCC	Personal continuity of care	10.1016/j.wombi.2022.03.004
**Alcaraz-Vidal**	2023	Spain	Exploring home births in Catalonia (Spain): A cross-sectional study of women's experiences and influencing factors	Homebirth	Place of birth - outside the obstetric unit	10.1111/jan.15989
**Alcaraz-Vidal**	2024	Spain	First alongside midwifery led unit in a high complexity public hospital in Spain: Maternal and neonatal outcomes	Birthcentre	Place of birth - outside the obstetric unit	10.1016/j.wombi.2024.01.003
**Allen**	2017	Australia	The motivation and capacity to go ‘above and beyond’: Qualitative analysis of free-text survey responses in the M@NGO randomised controlled trial of caseload midwifery	MLCC	Personal continuity of care	10.1016/j.midw.2017.03.012
**Allen**	2020	Australia	Women’s unmet needs in early labour: Qualitative analysis of free-text survey responses in the M@NGO trial of caseload midwifery	MLCC	Personal continuity of care	10.1016/j.midw.2020.102751
**Allen**	2019	Australia	The impact of caseload midwifery, compared with standard care, on women's perceptions of antenatal care quality: Survey results from the M@NGO randomized controlled trial for women of any risk	MLCC	Personal continuity of care	10.1111/birt.12436
**Allen**	2015	Australia	Does model of maternity care make a difference to birth outcomes for young women? A retrospective cohort study	MLCC	Personal continuity of care	10.1016/j.ijnurstu.2015.04.011

First author	Year	Country	Title	Element of Organisation	Group of elements of organisation	DOI
**Allen**	2016	Australia	How optimal caseload midwifery can modify predictors for pretermbirth in young women: Integrated findings from a mixedmethods study	MLCC	Personal continuity of care	10.1016/j.midw.2016.07.012
**Allen**	2012	Review	Does the way maternity care is provided affect maternal and neonatal outcomes for young women? A review of the research literature	Shared care (explicit shared interprofessional responsibility for the client)	Interventions to enhance inter-disciplinary collaboration	10.1016/j.wombi.2011.03.002
**Alliman**	2016	Review	Maternal Outcomes in Birth Centers: An Integrative Review of the Literature	Birthcentre	Place of birth - outside the obstetric unit	10.1111/jmwh.12356
**Altman**	2017	USA	The Cost of Nurse-Midwifery Care: Use of Interventions, Resources, and Associated Costs in the Hospital Setting	MLCC	Personal continuity of care	10.1016/j.whi.2017.01.002
**Altman**	2022	USA	Factors associated with person-centered care during pregnancy and birth for Black women and birthing people in California	Supportive care	Woman centred care	10.1111/birt.12675
**Andersson**	2012	Sweden	Parents' experiences and perceptions of group-based antenatal care in four clinics in Sweden	Group antenatal care	Care by a midwife	10.1016/j.midw.2011.07.006
**Andersson**	2013	Sweden	Mothers' satisfaction with group antenatal care versus individual antenatal care – A clinical trial	Group antenatal care	Care by a midwife	10.1016/j.srhc.2013.08.002
**Anyiam**	2024	USA	Listening to Black Women's Perspectives of Birth Centers and Midwifery Care: Advocacy, Protection, and Empowerment	Birthcentre	Place of birth - outside the obstetric unit	10.1111/jmwh.13635

First author	Year	Country	Title	Element of Organisation	Group of elements of organisation	DOI
**Aquino**	2018	England	A focus group study of women's views and experiences of maternity care as delivered collaboratively by midwives and health visitors in England	Shared care (explicit shared interprofessional responsibility for the client)	Interventions to enhance inter-disciplinary collaboration	10.1186/s12884-018-2127-0
**Augur**	2022	USA	The Early Care Model for Initiation of Perinatal Care: "I Actually Felt Listened To"	Supportive care	Woman centred care	10.1111/jmwh.13435
**Aune**	2014	Norway	Is a midwife's continuous presence during childbirth a matter of course? Midwives' experiences and thoughts about factors that may influence their continuous support of women during labour	Continous support during labour	Care by a midwife	10.1016/j.midw.2013.02.001
**Aune**	2021	Norway	Norwegian midwives' experiences of relational continuity of midwifery care in the primary healthcare service: A qualitative descriptive study	MLCC	Personal continuity of care	10.1177/20571585209732 02
**Aune**	2021	Norway	Early discharge from hospital after birth: How Norwegian parents experience postnatal home visits by midwives – A qualitative study	Midwife led post natal care	Care by a midwife	10.1016/j.srhc.2021.100672
**Avalos**	2024	USA	Group Multimodal Prenatal Care and Postpartum Outcomes	Group antenatal care	care by a midwife	10.1001/jamanetworkopen.2024.12280
**Barclay**	2012	Australia	Improving Aboriginal maternal and infant health services in the Top End' of Australia; synthesis of the findings of a health services research program aimed at engaging stakeholders, developing research capacity and embedding change	MLCC	Personal continuity of care	10.1186/1472-6963-14-241

First author	Year	Country	Title	Element of Organisation	Group of elements of organisation	DOI
**Barr**	2024	Australia	Perinatal continuity of care for mothers with depressive symptoms: perspectives of mothers and clinicians	MLCC	Personal continuity of care	10.3389/fpsyt.2024.1385120
**Bartuseviciene**	2018	Lithuania	Comparison of midwife-led and obstetrician-led care in Lithuania: A retrospective cohort study	Midwife led intrapartum care	Care by a midwife	10.1016/j.midw.2018.06.017
**Beake**	2013	England	Caseload midwifery in a multi-ethnic community: The women's experiences	MLCC	Personal continuity of care	10.1016/j.midw.2013.01.003
**Berge**	2020	USA	Using an interprofessional prenatal group care model to address disparities in pregnancy-related outcomes in a high risk population in a family medicine residency clinic	Group antenatal care	Care by a midwife	10.1016/j.xjep.2019.100300
**Bernitz**	2016	Norway	Evaluation of satisfaction with care in a midwifery unit and an obstetric unit: a randomized controlled trial of low-risk women	Birthcentre	Place of birth - outside the obstetric unit	10.1186/s12884-016-0932-x
**Bo geboortezorg en samenwerkende kraamorganisaties**	2020	The Netherlands	Kraamzorg op maat de nieuwe manier van indiceren	Shared decision making	Woman centred care	Unpublished access through https://bogeboortezorg.nl/
**Bodner**	2017	Austria	A ten-year study of midwife-led care at an Austrian tertiary care center: a retrospective analysis with special consideration of perineal trauma	Midwife led intrapartum care	Care by a midwife	10.1186/s12884-017-1544-9.

First author	Year	Country	Title	Element of Organisation	Group of elements of organisation	DOI
**Bohren**	2017	Review	Continuous support for women during childbirth	Continous support during labour	Care by a midwife	10.1002/14651858.CD003766.pub6
**Bonvicini**	2014	Italy	Public and private pregnancy care in Reggio Emilia Province: an observational study on appropriateness of care and delivery outcomes	Private care obstetrician	Personal continuity of care	10.1186/1471-2393-14-72
**Bradford**	2024	Australia	Diabetes in pregnancy: Women's views of care in a multi-ethnic, low socioeconomic population with midwifery continuity-of-care	MLCC	Personal continuity of care	10.1016/j.wombi.2024.01.005
**Brigante**	2023	UK	"She was there all the time". A qualitative study exploring how women at higher risk for preterm birth experience midwifery continuity of care	MLCC	Personal continuity of care	10.1016/j.wombi.2023.01.003
**Brock**	2014	Review	Identification and evaluation of models of antenatal care in Australia – A review of the evidence	MLCC	Personal continuity of care	10.1111/ajo.12210
**Buikencollectief**	2023	The Netherlands	Visie lokale en regionale cliëntenparticipatie in de geboortezorg	Integrating womens voices	Woman centred care	https://hetbuikencollectief.nl/app/uploads/2024/05/Visie-regionale-clientenparticipatie-versie-02-10-2023-DEFINITIEF.pdf

First author	Year	Country	Title	Element of Organisation	Group of elements of organisation	DOI
**Buultjens**	2021	Review	The contribution of group prenatal care to maternal psychological health outcomes: A systematic review	Group antenatal care	Care by a midwife	10.1016/j.wombi.2020.12.004
**Callander**	2021	Australia	Cost-effectiveness of public caseload midwifery compared to standard care in an Australian setting: a pragmatic analysis to inform service delivery	MLCC	Personal continuity of care	10.1093/intqhc/mzab084
**Callander**	2024	Australia	Continuity of care by a primary midwife (caseload midwifery): a cost analysis using results from the COSMOS randomised controlled trial	MLCC	Personal continuity of care	10.1136/gocm-2024-000008
**Callander**	2022	Australia	Using epidemiological and health economic measures to inform maternity staffing decisions: A guide	MLCC	Personal continuity of care	10.1016/j.wombi.2021.12.001
**Carrol**	2022	Ireland	‘Labour Hopscotch’: Women’s evaluation of using the steps during labor	Supportive care	Woman centred care	10.18332/ejm/152492
**Carroll**	2018	USA	Effects of episode-based payment on health care spending and utilization: Evidence from perinatal care in Arkansas	Bundled payment	Alternative payment model (other than fee for service)	10.1016/j.jhealeco.2018.06.010
**Caughey**	2019	Commentary	Home and Birth Center Birth in the United States	Homebirth	Place of birth - outside the obstetric unit	10.1097/AOG.0000000000003215
**Cellissen**	2022	The Netherlands	Integrating women’s voices in quality improvement for maternity care: A qualitative study	Integrating womens voices	Woman centred care	10.18332/ejm/152253
**Chaillet**	2015	Canada	A Cluster-Randomized Trial to Reduce Cesarean Delivery Rates in Quebec	Multidisciplinairy consultations and care pathways	Interventions to enhance inter-disciplinary collaboration	10.1056/NEJMoa1407120

First author	Year	Country	Title	Element of Organisation	Group of elements of organisation	DOI
**Chin**	2023	Sweden	Extended prenatal and postnatal home visits in a vulnerable area in Sweden—a pilot study	Supportive care	Woman centred care	10.1080/02813432.2023.2277756
**Cibralic**	2023	review	The impact of midwifery continuity of care on maternal mental health: A narrative systematic review	MLCC	Personal continuity of care	10.1016/j.midw.2022.103546
**Coker**	2023	USA	Community HealthWorkers in Early ChildhoodWell-Child Care for Medicaid-Insured Children A Randomized Clinical Trial	Shared care (explicit shared interprofessional responsibility for the client)	Interventions to enhance inter-disciplinary collaboration	10.1001/jama.2023.7197
**Combellick**	2023	USA	Midwifery care during labor and birth in the United States	Midwife led intrapartum care	Care by a midwife	10.1016/j.ajog.2022.09.044
**Cousins**	2022	Australia	Birthing on Country: improving maternal care in Australia	Supportive care	Woman centred care	10.1016/S0140-6736(23)00089-2
**Crocket**	2019	USA	Effects of a Multi-site Expansion of Group Prenatal Care on Birth Outcomes	Group antenatal care	Care by a midwife	10.1007/s10995-019-02795-4
**Crockett**	2022	USA	Group vs traditional prenatal care for improving racial equity in preterm birth and low birthweight: the Centering and Racial Disparities randomized clinical trial study	Group antenatal care	Care by a midwife	10.1016/j.ajog.2022.06.066
**Cross Barnet**	2022	USA	Limits of prenatal care coordination for improving birth outcomes among Medicaid participants	Cultural informed community care	Woman centred care	10.1016/j.ypmed.2022.107240

First author	Year	Country	Title	Element of Organisation	Group of elements of organisation	DOI
**Cummins**	2021	Australia	Exploring the value and acceptability of an antenatal and postnatal midwifery continuity of care model to women and midwives, using the Quality Maternal Newborn Care Framework	MLCC	Personal continuity of care	10.1016/j.wombi.2021.03.006
**Cummins**	2024	Australia	Midwifery continuity of care for women with perinatal mental health conditions: A cohort study from Australia	MLCC	Personal continuity of care	10.1111/birt.12838
**Cummins**	2023	Australia	Does midwifery continuity of care make a difference to women with perinatal mental health conditions: A cohort study, from Australia	MLCC	Personal continuity of care	10.1016/j.wombi.2022.08.002
**Cunningham**	2017	USA	Group prenatal care attendance: determinants and relationship with care satisfaction	Group antenatal care	Care by a midwife	10.1007/s10995-016-2161-3
**Cunningham**	2021	Review	Group Medical Care: A Systematic Review of Health Service Performance	Group antenatal care	Care by a midwife	10.3390/ijerph182312726
**Cusack**	2021	Review	Experiences of women discharged early following vaginal birth: a qualitative systematic review	Midwife led post natal care	Care by a midwife	10.11124/JBISRIR-D-19-00421
**Cutajar**	2023	Review	Model of care matters: An integrative review	Shared decision making	Woman centred care	10.1016/j.wombi.2022.12.007
**Dahl**	2020	Review	From Midwife-Dominated to Midwifery-Led Antenatal Care: A Meta-Ethnography	Midwife led antenatal care	Care by a midwife	10.3390/ijerph17238946
**Dahlberg (Norway)**	2016	Norway	Women's experiences of home visits by midwives in the early postnatal period	MLCC	Personal continuity of care	10.1016/j.srhc.2015.08.001

**First author**	**Year**	**Country**	**Title**	**Element of Organisation**	**Group of elements of organisation**	**DOI**
**Danhausen**	2022	USA	Strengthening Interprofessional Collaboration to Improve Transfers Between a Freestanding Birth Center and an Academic Medical Center	Shared care (explicit shared interprofessional responsibility for the client)	Interventions to enhance inter-disciplinary collaboration	10.1111/jmwh.13437
**Darling**	2021	Review	Facilitators and barriers to the implementation of a physiological approach during labour and birth: A systematic review and thematic synthesis	Supportive care	Woman centred care	10.1016/j.midw.2020.102861
**Darling**	2023	Canada	The impact of funding models on the integration of Ontario midwives: a qualitative study	Salary based midwifery funding	Alternative payment model (other than fee for service)	10.1186/s12913-023-10104-7
**Dawson**	2018	Australia	Comparing caseload and non-caseload midwives' burnout levels and professional attitudes: A national, cross-sectional survey of Australian midwives working in the public maternity system	MLCC	Personal continuity of care	10.1016/j.midw.2018.04.026
**de Jonge**	2015	The Netherlands	Severe Adverse Maternal Outcomes among Women in Midwife-Led versus Obstetrician-Led Care at the Onset of Labour in the Netherlands: A Nationwide Cohort Study	Homebirth	Place of birth - outside the obstetric unit	10.1371/journal.pone.0126266
**de Jonge**	2013	The Netherlands	Severe adverse maternal outcomes among low risk women with planned home versus hospital births in the Netherlands: nationwide cohort study	Homebirth	Place of birth - outside the obstetric unit	10.1136/bmj.f3263
**de Jonge**	2014	The Netherlands	Continuity of care: what matters to women when they are referred from primary to secondary care during labour? a qualitative interview study in the Netherlands	MLCC	Personal continuity of care	10.1186/1471-2393-14-103

First author	Year	Country	Title	Element of Organisation	Group of elements of organisation	DOI
**de Jonge**	2014	The Netherlands	Perinatal mortality and morbidity up to 28 days after birth among 743 070 low-risk planned home and hospital births: a cohort study based on three merged national perinatal databases	Homebirth	Place of birth - outside the obstetric unit	10.1111/1471-0528.13084
**de Vries**	2021	Review	A Scoping Review of Alternative Payment Models in Maternity Care: Insights in Key Design Elements and Effects on Health and Spending	Alternative payment model (other than fee for service)	Alternative payment model (other than fee for service)	10.5334/ijic.5535
**Denham**	2017	Scotland	Quality of care provided in two Scottish rural community maternity units: a retrospective case review	Birthcentre	Place of birth - outside the obstetric unit	10.1186/s12884-017-1374-9
**Digenis**	2020	Review	Reduced length of hospital stay after caesarean section: A systematic review examining women's experiences and psychosocial outcomes	Midwife led post natal care	Care by a midwife	10.1016/j.midw.2020.102855
**Dixon**	2023	New Zealand	Building positive respectful midwifery relationships: An analysis of women's experiences of continuity of midwifery care in Aotearoa New Zealand	MLCC	Personal continuity of care	10.1016/j.wombi.2023.06.008
**Doering**	2023	Japan	Seeking a connection: Women's lived experience of the woman-midwife relationship in mainstream maternity services in Japan	MLCC	Personal continuity of care	10.1016/j.wombi.2023.05.007
**Donnelan**	2018	Review	Cost-effectiveness of continuity of midwifery care for women with complex pregnancy: a structured review of the literature	MLCC	Personal continuity of care	10.1186/s13561-018-0217-3
**Donnellan**	2020	Australia	Differential access to continuity of midwifery care in Queensland, Australia	MLCC	Personal continuity of care	10.1071/AH19264

First author	Year	Country	Title	Element of Organisation	Group of elements of organisation	DOI
**Eikemo**	2022	Sweden	Support during the postnatal period: Evaluating new mothers' and midwives' experiences of a new, coordinated postnatal care model in a midwifery clinic in Sweden	MLCC	Personal continuity of care	10.1111/scs.13103
**Ertok**	2015	England	Evaluation of a Pay for Performance Scheme in Maternity Care: The Commissioning Quality and Innovation Payment Framework in England	Pay for quality (performance)	Alternative payment model (other than fee for service)	10.1016/j.sbspro.2015.06.333
**Facchetti**	2024	Australia	Continuity obstetric care demonstrates greater vaginal birth after caesarean success	MLCC	Personal continuity of care	10.1111/ajo.13790
**Fahlbeck**	2022	Sweden	'A longing for a sense of security' – Women's experiences of continuity of midwifery care in rural Sweden: A qualitative study	MLCC	Personal continuity of care	10.1016/j.srhc.2022.100759
**Fenwick**	2018	Australia	The emotional and professional wellbeing of Australian midwives: A comparison between those providing continuity of midwifery care and those not providing continuity	MLCC	Personal continuity of care	10.1016/j.wombi.2017.06.013
**Fernandez**	2021	Review	A realist review to explore how midwifery continuity of care may influence preterm birth in pregnant women	MLCC	Personal continuity of care	10.1111/birt.12547
**Fernandez Turienzo**	2023	UK	A continuity of care programme for women at risk of preterm birth in the UK: Process evaluation of a hybrid randomised controlled pilot trial	MLCC	Personal continuity of care	10.1371/journal.pone.0279695
**Ferrazzi**	2015	Italy	The outcome of midwife-led labor in low-risk women within an obstetric referral unit	Midwife led intrapartum care	Care by a midwife	10.3109/14767058.2014.958995

First author	Year	Country	Title	Element of Organisation	Group of elements of organisation	DOI
**Fong**	2024	Review	Effectiveness and implementation of lower-intensity weight management interventions delivered by the non-specialist workforce in postnatal women: a mixed-methods systematic review	Supportive care	Woman centred care	10.3389/fpubh.2024.1359680
**Ford**	2023	Review	Midwives' experience of personal/professional risk when providing continuity of care to women who decline recommendations: A meta-synthesis of qualitative studies	MLCC	Personal continuity of care	10.1016/j.wombi.2022.06.014
**Fox**	2023	Review	Midwifery continuity of care for women with complex pregnancies in Australia: An integrative review	MLCC	Personal continuity of care	10.1016/j.wombi.2022.07.001
**Fox**	2023	Review	Midwifery continuity of care for women with complex pregnancies in Australia: An integrative review	MLCC	Personal continuity of care	10.1016/j.wombi.2022.07.001
**Froh**	2022	USA	Lactation Outcomes After Participation in a Tailored Prenatal Nutrition Consultation Among Women With Infants With Congenital Anomalies	Supportive care	Woman centred care	10.1016/j.jogn.2022.07.007
**Frosig Pedersen**	2021	Review	Interventions to reduce preterm birth in pregnant women with psychosocial vulnerability factors-A systematic review	Group antenatal care	Care by a midwife	10.1016/j.midw.2021.103018
**Gao**	2014	Australia	A cost-consequences analysis of a Midwifery Group Practice for Aboriginal mothers and infants in the Top End of the Northern Territory, Australia	MLCC	Personal continuity of care	10.1016/j.midw.2013.04.004
**Gao**	2023	Australia	Birthing on country service compared to standard care for First Nations Australians: a cost-effectiveness analysis from a health system perspective	MLCC	Personal continuity of care	10.1016/j.lanwpc.2023.100722

First author	Year	Country	Title	Element of Organisation	Group of elements of organisation	DOI
**Gidaszewski**	2019	Australia	Comparison of the effect of caseload midwifery program and standard midwifery-led care on primiparous birth outcomes: A retrospective cohort matching study	MLCC	Personal continuity of care	10.1016/j.midw.2018.10.010
**Gillen**	2023	Review	Systematic review of women's experiences of planning home birth in consultation with maternity care providers in middle to high-income countries	Homebirth	Place of birth - outside the obstetric unit	10.1016/j.midw.2023.103733
**Gillen**	2023	Review	Systematic review of women's experiences of planning home birth in consultation with maternity care providers in middle to high-income countries	Homebirth	Place of birth - outside the obstetric unit	10.1016/j.midw.2023.103733
**Gregory**	2023	Ireland	"It could not have been more different." Comparing experiences of hospital-based birth and homebirth in Ireland: A mixed-methods survey	Homebirth	Place of birth - outside the obstetric unit	10.1016/j.wombi.2023.02.003
**Grigg**	2017	New Zealand	Evaluating Maternity Units: a prospective cohort study of freestanding midwife-led primary maternity units in New Zealand—clinical outcomes	Birthcentre	Place of birth - outside the obstetric unit	10.1136/bmjopen-2017-016288
**Grunebaum**	2022	USA	The impact of birth settings on pregnancy outcomes in the United States	Homebirth	Place of birth - outside the obstetric unit	10.1016/j.ajog.2022.08.011
**Haggsgard**	2024	Sweden	Impact of collegial midwifery assistance during second stage of labour on women's experience: a follow-up from the Swedish Oneplus randomised controlled trial	Midwife led intrapartum care	Care by a midwife	10.1136/bmjopen-2023-077458
**Haines**	2015	Australia	Continuity of midwifery care for rural women through caseload group practice: Delivering for almost 20 years	MLCC	Personal continuity of care	10.1111/ajr.12232

First author	Year	Country	Title	Element of Organisation	Group of elements of organisation	DOI
**Halfdansdottir**	2023	Iceland	Normal birth rates before and after the merging of mixed-risk and low-risk maternity wards in Iceland: A retrospective cohort study on the impact of inter-professional preventative measures	Shared care (explicit shared interprofessional responsibility for the client)	Interventions to enhance inter-disciplinary collaboration	10.1111/birt.12776
**Hanley**	2022	Review	Job satisfaction and sustainability of midwives working in caseload models of care: An integrative literature review	MLCC	Personal continuity of care	10.1016/j.wombi.2021.06.003
**Hans**	2018	USA	Randomized Controlled Trial of Doula-Home-Visiting Services: Impact on Maternal and Infant Health	Continous support by a doula	Personal continuity of care	10.1007/s10995-018-2537-7
**Harmsen**	2021	The Netherlands	Development of a Blueprint for Integrated Care for Vulnerable Pregnant Women	Multidisciplinairy consultations and care pathways	Interventions to enhance inter-disciplinary collaboration	10.1007/s10995-021-03340-y
**Hartz**	2012	Discussion	Australian caseload midwifery: The exception or the rule	MLCC	Personal continuity of care	10.1016/j.wombi.2011.01.001
**Hays**	2022	USA	Smooth Transitions: Enhancing Interprofessional Collaboration when Planned Community Births Transfer to Hospital Care	Shared care (explicit shared interprofessional responsibility for the client)	Interventions to enhance inter-disciplinary collaboration	10.1111/jmwh.13441
**Heberlein**	2016	USA	Qualitative Comparison of Women’s Perspectives on the Functions and Benefits of Group and Individual Prenatal Care	Group antenatal care	Care by a midwife	10.1111/jmwh.12379
**Heberlein**	2024	USA	Birth Outcomes for Medically High-Risk Pregnancies: Comparing Group to Individual Prenatal Care	Group antenatal care	Care by a midwife	10.1055/a-1682-2704

First author	Year	Country	Title	Element of Organisation	Group of elements of organisation	DOI
**Hildingsson**	2019	Sweden	A known midwife can make a difference for women with fear of childbirthbirth outcome and women's experiences of intrapartum care	MLCC	Personal continuity of care	10.1016/j.srhc.2019.06.004
**Hildingsson**	2020	Sweden	A continuity of care project with two on-call schedules: Findings from a rural area in Sweden	MLCC	Personal continuity of care	10.1016/j.srhc.2020.100551
**Hildingsson**	2020	Sweden	Birth outcome in a caseload study conducted in a rural area of Sweden-a register based study	MLCC	Personal continuity of care	10.1016/j.srhc.2020.100509
**Hildingsson**	2021	Sweden	Childbirth experience in women participating in a continuity of midwifery care project	MLCC	Personal continuity of care	10.1016/j.wombi.2020.04.010
**Hildingsson**	2021	Sweden	Quality of intrapartum care assessed by women participating in a midwifery model of continuity of care	MLCC	Personal continuity of care	10.18332/ejm/134502
**Hildingsson**	2021	Sweden	Women's Experiences of Care During Pregnancy in a Continuity of Midwifery Care Project in Rural Sweden	MLCC	Personal continuity of care	10.1016/j.srhc.2022.100759
**Hildingsson**	2023	Sweden	'A perfect fit' – Swedish midwives' interest in continuity models of midwifery care	MLCC	Personal continuity of care	10.1016/j.wombi.2022.04.014
**Hitzert**	2016	The Netherlands	Experiences of women who planned birth in a birthcentre compared to alternative planned places of birth. Results of the Dutch Birth Centre Study	Birthcentre	Place of birth – outside the obstetric unit	10.1016/j.midw.2016.06.004
**Hodgson**	2017	Canada	An evaluation of Interprofessional group antenatal care: a prospective comparative study	Group antenatal care	Care by a midwife	10.1186/s12884-017-1485-3

First author	Year	Country	Title	Element of Organisation	Group of elements of organisation	DOI
**Homer**	2016	Review	Models of maternity care: evidence for midwifery continuity of care	MLCC	Personal continuity of care	10.5694/mja16.00844
**Homer**	2017	England	Midwifery continuity of care in an area of high socio-economic disadvantage in London: A retrospective analysis of Albany Midwifery Practice outcomes using routine data	MLCC	Personal continuity of care	10.1016/j.midw.2017.02.009
**Hu**	2024	Australia	A cost analysis of upscaling access to continuity of midwifery carer: Population-based microsimulation in Queensland, Australia	MLCC	Personal continuity of care	10.1016/j.midw.2024.103998
**Hu**	2024	Australia	The financial impact of offering publicly funded homebirths: A population-based microsimulation in Queensland, Australia	Homebirth	Place of birth - outside the obstetric unit	10.1016/j.wombi.2023.07.129
**Hunter**	2019	UK	Better together: A qualitative exploration of women's perceptions and experiences of group antenatal care	Group antenatal care	Care by a midwife	10.1016/j.wombi.2018.09.001
**Hutton**	2015	Canada	Outcomes associated with planned place of birth among women with low-risk pregnancies	Homebirth	Place of birth - outside the obstetric unit	10.1503/cmaj.150564
**Ichikawa**	2015	Japan	Effectiveness of Home Visits in Pregnancy as a Public Health Measure to Improve Birth Outcomes	Supportive care	Woman centred care	10.1371/journal.pone.0137307
**Iida**	2021	Japan	Women's experience of receiving team-midwifery care in Japan: A qualitative descriptive study	MLCC	Personal continuity of care	10.1016/j.wombi.2020.09.020
**Isaline**	2019	Belgium	An exploratory cost-effectiveness analysis: Comparison between a midwife-led birth unit and a standard obstetric unit within the same hospital in Belgium	Midwife led intrapartum care	Care by a midwife	10.1016/j.midw.2019.05.004

First author	Year	Country	Title	Element of Organisation	Group of elements of organisation	DOI
**Israel**	2022	USA	Integrating Community Health Workers and Nurse Midwives on the Health-Care Team to Improve Birth and Breastfeeding Outcomes	Shared care (explicit shared interprofessional responsibility for the client)	Interventions to enhance inter-disciplinary collaboration	10.1891/JPE-2022-0025
**Janke**	2024	Germany	Women's needs and expectations in midwifery care - Results from the qualitative MiCa (midwifery care) study. Part 1: Preconception and pregnancy	Midwife led antenatal care	Care by a midwife	10.1016/j.heliyon.2024.e25862
**Janssen**	2015	Canada	Costs of Planned Home vs. Hospital Birth in British Columbia Attended by Registered Midwives and Physicians	Homebirth	Place of birth - outside the obstetric unit	10.1371/journal.pone.0133524
**Jepsen**	2018	Danmark	Labour outcomes in caseload midwifery and standard care: a register-based cohort study	MLCC	Personal continuity of care	10.1186/s12884-018-2090-9
**Johansson**	2019	Sweden	Mothers´experiences in relation to a new Swedish postnatal home-based model of midwifery care–A cross-sectional study	Midwife led post natal care	Care by a midwife	10.1016/j.midw.2019.07.010
**Johansson**	2022	Sweden	Women's experience of the decision making process for home based postnatal midwifery care when discharged early from hospital; a swedish interview study	Midwife led post natal care	Care by a midwife	10.18332/ejm/152547
**Johri**	2017	Canada	A cluster-randomized trial to reduce caesarean delivery rates in Quebec: costeffectiveness analysis	Multidisciplinairy consultations and care pathways	Interventions to enhance inter-disciplinary collaboration	10.1186/s12916-017-0859-8
**Jolles**	2022	USA	Strong Start Innovation: Equitable Outcomes Across Public and Privately Insured Clients Receiving Birth Center Care	Birthcentre	Place of birth - outside the obstetric unit	10.1111/jmwh.13439

First author	Year	Country	Title	Element of Organisation	Group of elements of organisation	DOI
**Kanda**	2022	Review	Parent satisfaction with sustained home visiting care for mothers and children: an integrative review	Midwife led post natal care	Care by a midwife	10.1186/s12913-022-07666-3
**Kashani**	2020	Australia	Caseload midwifery in a rural Australian setting: A qualitative descriptive study	MLCC	Personal continuity of care	10.18332/ejm/131240
**Kemp**	2012	Australia	Benefits of psychosocial intervention and continuity of care by child and family health nurses in the pre- and postnatal period: process evaluation	Supportive care	Woman centred care	10.1111/jan.12052
**Kenny**	2015	Ireland	A cost-comparison of midwife-led compared with consultant-led maternity care in Ireland (theMidUstudy)	MLCC	Personal continuity of care	10.1016/j.midw.2015.06.012
**Khan**	2023	Review	Targeted health and social care interventions for women and infants who are disproportionately impacted by health inequalities in high-income countries: a systematic review	Supportive care	Woman centred care	10.1186/s12939-023-01948-w
**Kildea**	2016	Australia	Remote links: Redesigning maternity care for Aboriginal women from remote communities in Northern Australia – A comparative cohort study	MLCC	Personal continuity of care	10.1016/j.midw.2016.01.009
**Kloester**	2023	Australia	How midwives facilitate informed decisions in the third stage of labour – an exploration through portraiture	Shared decision making	Woman centred care	10.1016/j.midw.2023.103868
**Knape**	2014	Germany	The association between attendance of midwives and workload of midwives with the mode of birth: secondary analyses in the German healthcare system	Midwife led intrapartum care	Care by a midwife	10.1186/1471-2393-14-300
**Kozhimannil**	2018	USA	Cesarean Delivery Rates and Costs of Childbirth in a State Medicaid Program After Implementation of a Blended Payment Policy	Bundled payment	Alternative payment model (other than fee for service)	10.1097/MLR.0000000000000937

First author	Year	Country	Title	Element of Organisation	Group of elements of organisation	DOI
**Kurth**	2016	Switzerland	Safe start at home: what parents of newborns need after early discharge from hospital – a focus group study	Midwife led post natal care	Care by a midwife	10.1186/s12913-016-1300-2
**Lagendijk**	2018	The Netherlands	Antenatal non-medical risk assessment and care pathways to improve pregnancy outcomes: a cluster randomised controlled trial	Multidisciplinairy consultations and care pathways	Interventions to enhance inter-disciplinary collaboration	10.1007/s10654-018-0387-7
**Lai**	2024	Australia	Outcomes and perspectives of woman-newborn dyads following discharge from a quaternary maternity service in Australia: A cross-sectional survey	Midwife led post natal care	Care by a midwife	10.1016/j.wombi.2023.10.007
**Laliberte**	2016	Canada	A Randomized Controlled Trial of Innovative Postpartum Care Model for Mother-Baby Dyads	Supportive care	Woman centred care	10.1371/journal.pone.0148520
**Larsson**	2021	Sweden	Demanding and rewarding: Midwives experiences of starting a continuity of care project in rural Sweden	MLCC	Personal continuity of care	10.18332/ejm/133573
**Lata**	2019	Poland	Comparison of demographic factors and selected maternity care variables in women who chose midwife-led or obstetrician-led antenatal care respectively	Midwife led antenatal care	Care by a midwife	10.2478/pielxxiw-2019-0002
**Lazar**	2021	Review	A systematic review of providers' experiences of facilitating group antenatal care	Group antenatal care	Care by a midwife	10.1186/s12978-021-01200-0
**Levorstad**	2022	Norway	Midwives' experiences of an organizational change in early postpartum care services in Norway: A qualitative study	Midwife led post natal care	Care by a midwife	10.18332/ejm/147746
**Lewis**	2020	UK	Midwives' Experience of Providing Continuity of Care in a Pilot Project: Findings of a Prospective Qualitative Research Study	MLCC	Personal continuity of care	10.1891/IJCBIRTH-D-20-00009

First author	Year	Country	Title	Element of Organisation	Group of elements of organisation	DOI
**Loewenberg**	2017	USA	Midwives as prenatal care providers in the United States	Midwife led care - whole carepath	Care by a midwife	Loewenberg Weisband J. Midwives as prenatal care providers in the United States (Doctoral dissertation, The Ohio State University).
**Lundborg**	2024	Sweden	Midwifery Continuity of Care During Pregnancy, Birth, and the Postpartum Period: A Matched Cohort Study	MLCC	Personal continuity of care	10.1111/birt.12875
**Lundborg**	2024	Sweden	Midwifery Continuity of Care During Pregnancy, Birth, and the Postpartum Period: A Matched Cohort Study	MLCC	Personal continuity of care	10.1111/birt.12875
**Lundgren**	2020	Sweden	Health professionals' perceptions of a midwifery model of woman-centred care implemented on a hospital labour ward	Supportive care	Woman centred care	10.1016/j.wombi.2019.01.004
**Lutenbacher**	2013	USA	Does Additional Prenatal Care in the Home Improve Birth Outcomes for Women with a Prior Preterm Delivery? A Randomized Clinical Trial	Midwife led antenatal care	Care by a midwife	10.1007/s10995-013-1344-4
**Lyndon**	2016	USA	Thematic analysis of US stakeholder views on the influence of labour nurses' care on birth outcomes	Supportive care	Woman centred care	10.1136/bmjqs-2016-005859

First author	Year	Country	Title	Element of Organisation	Group of elements of organisation	DOI
**Maillefer**	2015	Switzerland	Women and healthcare providers' perceptions of a midwife-led unit in a Swiss university hospital: a qualitative study	MLCC	Personal continuity of care	10.1186/s12884-015-0477-4
**Makarova**	2024	Germany	Women's expectations, preferences and needs in midwifery care – results from the qualitative Midwifery Care (MiCa) study: Childbirth and early parenthood	Midwife led care - whole carepath	Care by a midwife	10.1016/j.midw.2024.103990
**Malouf**	2018	Review	Expectations and experiences of hospital postnatal care in the UK: a systematic review of quantitative and qualitative studies	Midwife led post natal care	Care by a midwife	10.1136/bmjopen-2018-022212
**Martin**	2015	Australia	Midwives' experiences of working in a new service delivery model: the next birth after caesarean service	Midwife led antenatal care	Care by a midwife	Martin T, Hauck Y, Fenwick J, Butt J, Wood J. (2015) Midwives' experiences of working in a new service delivery model: the next birth after caesarean service. Evidence Based

First author	Year	Country	Title	Element of Organisation	Group of elements of organisation	DOI
						Midwifery 13(1): 10-14
**Martin**	2023	Review	A systematic review of the cost-effectiveness of maternity models of care	Midwife led care - whole carepath	Care by a midwife	10.1186/s12884-023-06180-6
**Martin-Arribas**	2022	Spain	A comparison between midwifery and obstetric care at birth in Spain: Across-sectional study of perinatal outcomes	Midwife led care - whole carepath	Care by a midwife	10.1016/j.ijnurstu.2021.104129
**Mazzoni**	2017	Review	Group prenatal care	Group antenatal care	Care by a midwife	10.1016/j.ajog.2017.02.006
**McCalman**	2023	Australia	Exploring satisfaction among women having a First Nations baby at one of three maternity hospitals offering culturally specific continuity of midwife care in Victoria, Australia: A cross-sectional survey	MLCC	Personal continuity of care	10.1016/j.wombi.2023.06.003

First author	Year	Country	Title	Element of Organisation	Group of elements of organisation	DOI
**McCalman**	2024	Australia	"Safe, connected, supported in a complex system." Exploring the views of women who had a First Nations baby at one of three maternity services offering culturally tailored continuity of midwife care in Victoria, Australia. A qualitative analysis of free-text survey responses	MLCC	Personal continuity of care	10.1016/j.wombi.2024.01.009
**McCartthy**	2017	England	Midwifery continuity: The use of social media	MLCC	Personal continuity of care	10.1016/j.midw.2017.05.012
**McConnel**	2023	USA	Effect of an Intensive Nurse Home Visiting Program on Adverse Birth Outcomes in a Medicaid-Eligible Population A Randomized Clinical Trial	Supportive care	Woman centred care	10.1001/jama.2022.9703
**McCue**	2022	USA	Impact of a Community Health Worker (CHW) Home Visiting Intervention on Any and Adequate Prenatal Care Among Ethno-Racially Diverse Pregnant Women of the US Southwest	Supportive care	Woman centred care	10.1007/s10995-022-03506-2
**McFarland**	2020	Review	The experiences of midwives in integrated maternity care: A qualitative metasynthesis	Shared care (explicit shared interprofessional responsibility for the client)	Interventions to enhance inter-disciplinary collaboration	10.1016/j.midw.2019.102544
**McIntyre**	2012	Review	Safety of non-medically led primary maternity care models: a critical review of the international literature	Midwife led intrapartum care	Care by a midwife	10.1071/AH11039
**McKellar**	2023	Australia	'There is no other option': Exploring health care providers' experiences implementing regional multisite midwifery model of care in South Australia	MLCC	Personal continuity of care	10.1111/ajr.13066

First author	Year	Country	Title	Element of Organisation	Group of elements of organisation	DOI
**McLaughlin**	2023	Australia	Comparing the views of caseload midwives working with First Nations families in an all-risk, culturally responsive model with midwives working in standard caseload models, using a cross-sectional survey design	MLCC	Personal continuity of care	10.1016/j.wombi.2023.05.006
**McNelis**	2013	Ireland	Women's experiences of care during labour in a midwifery-led unit in the Republic of Ireland	Birthcentre	Place of birth - outside the obstetric unit	10.12968/bjom.2013.21.9.622
**McRae**	2018	Canada	Reduced prevalence of small-for gestational- age and preterm birth for women of low socioeconomic position: a population-based cohort study comparing antenatal midwifery and physician models of care	MLCC	Personal continuity of care	10.1136/bmjopen-2018-022220
**McRae**	2019	Canada	Improving birth outcomes for women who are substance using or have mental illness: a Canadian cohort study comparing antenatal midwifery and physician models of care for women of low socioeconomic position	MLCC	Personal continuity of care	10.1186/s12884-019-2428-y
**Meghea**	2023	USA	Community HealthWorker Home Visiting, Birth Outcomes, Maternal Care, and Disparities Among Birthing Individuals With Medicaid Insurance	Cultural informed community care	Woman centred care	10.1001/jamapediatrics.2023.2310
**Merz**	2020	Germany	Maternal and neonatal outcome of births planned in alongside midwifery units: a cohort study from a tertiary center in Germany	Continous support during labour	Care by a midwife	10.1186/s12884-020-02962-4
**Miller**	2022	Australia	A direct comparison of patient-reported outcomes and experiences in alternative models of maternity care in Queensland, Australia	Private care obstetrician	Personal continuity of care	10.1371/journal.pone.0271105
**Miller**	2022	Australia	A direct comparison of patient-reported outcomes and experiences in alternative models of maternity care in Queensland, Australia	Private care obstetrician	Personal continuity of care	10.1371/journal.pone.0271105

First author	Year	Country	Title	Element of Organisation	Group of elements of organisation	DOI
**Moncrief**	2018	Discussion	Can continuity bring birth back to women and normality back to midwives?	MLCC	Personal continuity of care	10.12968/bjom.2018.26.10.642
**Morr**	2021	Switzerland	Obstetrician involvement in planned midwife-led births: a cohort study in an obstetric department of a University Hospital in Switzerland	Midwife led intrapartum care	Care by a midwife	10.1186/s12884-021-04209-2
**Morrow**	2013	Australia	Redesigning postnatal care: exploring the views and experiences of midwives	Supportive care	Woman centred care	10.1016/j.midw.2011.11.006
**Mottl**	2023	USA	Effectiveness of an Enhanced Community Doula Intervention in a Safety Net Setting: A Randomized Controlled Trial	Cultural informed community care	Woman centred care	10.1089/heq.2022.0200
**Munns**	2016	Review	Effectiveness and experiences of families and support workers participating in peer-led parenting support programs delivered as home visiting programs: a comprehensive systematic review	Cultural informed community care	Woman centred care	10.11124/JBISRIR-2016-003166
**Murray**	2022	Canada	Promoting safety and role clarity among health professionals on Canada's First Alongside Midwifery Unit (AMU): A mixed-methods evaluation	MLCC	Personal continuity of care	10.1016/j.midw.2022.103366
**Murray**	2024	Canada	Comparing birth experiences and satisfaction with midwifery care before and after the implementation of Canada's first Alongside Midwifery Unit (AMU)	Birthcentre	Place of birth - outside the obstetric unit	10.1371/journal.pone.0306916
**Naginevic iute**	2023	Lithuania	Woman-Centered Care: Standardized Outcomes Measure	Integrating womens voices	Woman centred care	10.3390/medicina59091537

First author	Year	Country	Title	Element of Organisation	Group of elements of organisation	DOI
**Naughton**	2021	Review	Providing woman-centred care in complex pregnancy situations	Supportive care	Woman centred care	10.1016/j.midw.2021.103060
**Neerland**	2022	USA	Prenatal care in US birth centers: Midwives' perceptions of contributors to birthing People's confidence in physiologic birth	Midwife led antenatal care	Care by a midwife	10.1111/birt.12676
**Neppelenbroek**	2023	The Netherlands	Antenatal cardiotocography in primary midwife-led care: Women's satisfaction	Redistribution of care from medical specialist to community midwife	Care by a midwife	10.1111/birt.12725
**Neppelenbroek**	2024	The Netherlands	Antenatal cardiotocography in dutch primary midwife-led care: Maternal and perinatal outcomes and serious adverse events. A prospective observational cohort study	Redistribution of care from medical specialist to community midwife	Care by a midwife	10.1016/j.wombi.2023.08.006
**Newton**	2014	Australia	Comparing satisfaction and burnout between caseload and standard care midwives: findings from two cross-sectional surveys conducted in Victoria, Australia	MLCC	Personal continuity of care	10.1186/s12884-014-0426-7
**Newton**	2016	Australia	Understanding the 'work' of caseload midwives: A mixed-methods exploration of two caseload midwifery models in Victoria, Australia	MLCC	Personal continuity of care	10.1016/j.wombi.2015.10.011
**Newton**	2021	Australia	Midwives' views of caseload midwifery – comparing the caseload and non-caseload midwives' opinions. A cross-sectional survey of Australian midwives.	MLCC	Personal continuity of care	10.1016/j.wombi.2020.06.006

First author	Year	Country	Title	Element of Organisation	Group of elements of organisation	DOI
**Niles**	2023	USA	Examining respect, autonomy, and mistreatment in childbirth in the US: do provider type and place of birth matter?	Midwife led care - whole carepath	Care by a midwife	10.1186/s12978-023-01584-1
**Nilsson**	2019	Sweden	Midwives' care on a labour ward prior to the introduction of a midwifery model of care: a field of tension	Midwife led intrapartum care	Care by a midwife	10.1080/17482631.2019.1593037
**Norona**	2023	USA	Rates of Preterm Birth and Low Birth Weight in an Adolescent Obstetric Clinic: Achieving Health Equity Through Trauma-Informed Care	Shared care (explicit shared interprofessional responsibility for the client)	Interventions to enhance inter-disciplinary collaboration	10.1089/heq.2023.0075
**Nove**	2012	UK	Comparing the odds of postpartum haemorrhage in planned home birth against planned hospital birth: results of an observational study of over 500,000 maternities in the UK	Homebirth	Place of birth - outside the obstetric unit	10.1186/1471-2393-12-130
**Ny**	2023	Sweden	Does model of care affect women's health and wellbeing in the perinatal period in Sweden?	Midwife led antenatal care	Care by a midwife	bjom.2023.31.5.260
**Offerhaus**	2020	The Netherlands	Women's characteristics and care outcomes of caseload midwifery care in the Netherlands: a retrospective cohort study	MLCC	Personal continuity of care	10.1186/s12884-020-03204-3
**Pace**	2022	Review	Midwife experiences of providing continuity of carer: A qualitative systematic review	MLCC	Personal continuity of care	10.1016/j.wombi.2021.06.005
**Palau**	2023	Spain	The first alongside midwifery unit in Spain: A retrospective cohort study of maternal and neonatal outcomes	Birthcentre	Place of birth - outside the obstetric unit	10.1111/birt.12749

First author	Year	Country	Title	Element of Organisation	Group of elements of organisation	DOI
**Palau**	2024	Spain	Beyond the numbers: A phenomenological analysis of women's childbirth experiences in Spain's evolving healthcare system	Birthcentre	Place of birth - outside the obstetric unit	10.18332/ejm/191895
**Patel**	2013	UK	User engagement in the delivery and design of maternity services	Integrating womens voices	Woman centred care	10.1016/j.bpobgyn.2013.04.006
**Pelak**	2023	Australia	A content analysis of women's experiences of different models of maternity care: the Birth Experience Study (BESt)	Private care by midwife	Personal continuity of care	10.1186/s12884-023-06130-2
**Perdok**	2018	The Netherlands	Continuity of care is an important and distinct aspect of childbirth experience: findings of a survey evaluating experienced continuity of care, experienced quality of care and women's perception of labor	MLCC	Personal continuity of care	10.1186/s12884-017-1615-y
**Perez-Marinez**	2019	Spain	Postpartum complications in women attended by midwives instead of obstetricians	Midwife led post natal care	Care by a midwife	10.1016/j.midw.2019.04.009
**Permezel**	2015	Australia	Pregnancy outcome at term in low-risk population: Study at a tertiary obstetric hospital	Midwife led care - whole carepath	Care by a midwife	10.1111/jog.12695
**Perrella**	2022	Australia	Maternal Evaluation of a Team-Based Maternity Care Model for Women of Low Obstetric Risk	Shared care (explicit shared interprofessional responsibility for the client)	Interventions to enhance inter-disciplinary collaboration	10.1177/23743735221092606
**Perriman**	2018	Review	What women value in the midwifery continuity of care model: A systematic review with meta-synthesis	MLCC	Personal continuity of care	10.1016/j.midw.2018.04.011

First author	Year	Country	Title	Element of Organisation	Group of elements of organisation	DOI
**Pickelsimer**	2015	Commentary	Group Prenatal Care: Has Its Time Come?	Group antenatal care	Care by a midwife	10.1097/GRF.0000000000000095
**Pirwany**	2020	Canada	Impact of Provider Payment Structure on Obstetric Interventions and Outcomes: A Difference-in- Differences Analysis	Salary for medical specialist - gynacologist	Alternative payment model (other than fee for service)	10.1016/j.jogc.2019.11.071
**Poggianella**	2023	Italy	Women's experience of continuity of midwifery care in North-Eastern Italy: A qualitative study	MLCC	Personal continuity of care	10.18332/ejm/159358
**Poskiene**	2021	Lithuania	Comparison of vaginal birth outcomes in midwifery-led versus physician-led setting: A propensity score-matched analysis	MLCC	Personal continuity of care	10.1515/med-2021-0373
**Posthumus**	2013	Opinion	Bridging Between Professionals in Perinatal Care: Towards Shared Care in The Netherlands	Shared care (explicit shared interprofessional responsibility for the client)	Interventions to enhance inter-disciplinary collaboration	10.1007/s10995-012-1207-4
**Rayment**	2020	UK	Project 20: Midwives' insight into continuity of care models for women with social risk factors: what works, for whom, in what circumstances, and how	MLCC	Personal continuity of care	10.1016/j.midw.2020.102654
**Rayment**	2021	UK	Project20: Does continuity of care and community-based antenatal care improve maternal and neonatal birth outcomes for women with social risk factors? A prospective, observational study	MLCC	Personal continuity of care	10.1371/journal.pone.0250947
**Rayment**	2023	UK	Project20: Maternity care mechanisms that improve (or exacerbate) health inequalities. A realist evaluation	MLCC	Personal continuity of care	10.1016/j.wombi.2022.11.006

First author	Year	Country	Title	Element of Organisation	Group of elements of organisation	DOI
**Rayner**	2012	Australia	Care providers' views and experiences of postnatal care in private hospitals in Victoria, Australia	Midwife led post natal care	Care by a midwife	10.1016/j.midw.2012.05.006
**Reeve**	2016	Australia	Community outreach midwifery-led model improves antenatal access in a disadvantaged population	Shared care (explicit shared interprofessional responsibility for the client)	Interventions to enhance inter-disciplinary collaboration	10.1111/ajr.12249
**Reitsma**	2020	Review	Maternal outcomes and birth interventions among women who begin labour intending to give birth at home compared to women of low obstetrical risk who intend to give birth in hospital: A systematic review and meta-analyses	Homebirth	Place of birth - outside the obstetric unit	10.1016/j.eclinm.2020.100319
**Renfrew**	2014	Review	Midwifery and quality care: findings from a new evidence informed framework for maternal and newborn care	Supportive care	Woman centred care	10.1016/S0140-6736(14)60789-3
**Ricchi**	2019	Review	The midwifery-led care model: a continuity of care model in the birth path	MLCC	Personal continuity of care	10.23750/abm.v90i6-S.8621
**Roxburgh**	2021	Australia	Satisfaction with general practitioner obstetrician-led maternity care in rural Western Australia	Private care obstetrician	Personal continuity of care	10.1111/ajr.12783
**Rubashkin**	2021	Hungary	Examining obstetric interventions and respectful maternity care in Hungary: Do informal payments for continuity of care link to quality	Private care by midwife	Personal continuity of care	10.1111/birt.12540
**Rubashkin**	2024	Hungary	In search of respect and continuity of care: Hungarian women's experiences with midwifery-led, community birth	Private care by midwife	Personal continuity of care	10.1111/birt.12818

First author	Year	Country	Title	Element of Organisation	Group of elements of organisation	DOI
**Saetrum**	2023	Norway	User satisfaction with antenatal care in Norway	Midwife led antenatal care	Care by a midwife	10.1111/birt.12768
**Sakala**	2022	USA	Improving Our Maternity Care Now Through Community Birth Settings	Birthcentre	Place of birth - outside the obstetric unit	10.1891/JPE-2022-0015
**Sakala**	2022	USA	Improving Our Maternity Care Now Through Midwifery	MLCC	Personal continuity of care	10.1891/JPE-2022-0014
**Sandall**	2024	Review UPDATED	Midwife continuity of care models versus other models of care for childbearing women (Review)	MLCC	Personal continuity of care	10.1002/14651858.CD004667.pub6
**Sands**	2023	Review	Birth environments for women with complex pregnancies: A mixed-methods systematic review	Supportive care	Woman centred care	10.1016/j.wombi.2022.04.008
**Scheefhals**	2024	The Netherlands	Integrating Maternity Care Through Bundled Payments In The Netherlands: Early Results And Policy Lessons	Bundled payment	Alternative payment model (other than fee for service)	10.1377/hlthaff.2023.01637
**Schwind**	2023	Switzerland	"But at home, with the midwife, you are a person": experiences and impact of a new early postpartum home-based midwifery care model in the view of women in vulnerable family situations	Midwife led post natal care	Care by a midwife	10.1186/s12913-023-09352-4
**Shahshahani**	2024	Sweden	Midwifery continuity of care, breastfeeding and neonatal hyperbilirubinemia: A retrospective cohort study	MLCC	Personal continuity of care	10.1016/j.midw.2024.104079
**Sheeder**	2012	Review	A Review of Prenatal Group Care Literature: The Need for a Structured Theoretical Framework and Systematic Evaluation	Group antenatal care	Care by a midwife	10.1007/s10995-010-0709-1

First author	Year	Country	Title	Element of Organisation	Group of elements of organisation	DOI
**Shenton**	2024	Australia	Improving maternal and neonatal outcomes for women with gestational diabetes through continuity of midwifery care: A cross-sectional study	MLCC	Personal continuity of care	10.1016/j.wombi.2024.101597
**Shin**	2023	USA	Improving Birth Outcomes Among Low-Income Families: The Effect of a Home Visiting Intervention	Supportive care	Woman centred care	10.1177/00099228231158367
**Shipton**	2023	review	Midwife-Led Continuity of Antenatal Care and Breastfeeding Duration Beyond Postpartum Hospital Discharge: A Systematic Review	Midwife led antenatal care	Care by a midwife	10.1177/08903344221126644
**Shovlin**	2024	USA	U.S. Healthcare Provider Views and Practices Regarding Planned Birth Setting	Birthcentre	Place of birth – outside the obstetric unit	10.1086/728142
**Simmelink**	2025	The Netherlands	Comment on 'Outcome of induction of labour at 41 weeks with foley catheter in midwifery-led care' of Velthuijs et al	Redistribution of care from medical specialist to community midwife	Care by a midwife	10.1016/j.midw.2025.104369
**Sivertsen**	2020	Review	Aboriginal and Torres Strait Islander family access to continuity of health care services in the first 1000 days of life: a systematic review of the literature	MLCC	Personal continuity of care	10.1186/s12913-020-05673-w
**Sivertsen**	2022	Australia	A call for culture-centred care: exploring health workers' perspectives of positive care experiences and culturally responsive care provision to Aboriginal women and their infants in mainstream health in South Australia	Cultural informed community care	Woman centred care	10.1186/s12961-022-00936-w
**Sorbara**	2024	Canada	Postpartum Emergency Department Use Following Midwifery-Model vs Obstetrics-Model Care	Midwife led intrapartum care	Care by a midwife	10.1001/jamanetworkopen.2024.8676

First author	Year	Country	Title	Element of Organisation	Group of elements of organisation	DOI
**Souter**	2019	USA	Comparison of Midwifery and Obstetric Care in Low-Risk Hospital Births	MLCC	Personal continuity of care	10.1097/AOG.0000000000003521
**Souto**	2022	Review	Midwives' interventions for reducing fear of childbirth in pregnant women: a scoping review	Midwife led antenatal care	Care by a midwife	10.11124/JBIES-21-00382
**Stapleton**	2013	USA	Outcomes of Care in Birth Centers: Demonstration of a Durable Model	MLCC	Personal continuity of care	10.1111/jmwh.12003
**Stas**	2023	Belgium	Maternal satisfaction with reduced postnatal length of stay in Brussels: evidence from the KOZI&Home program	Midwife led post natal care	Care by a midwife	10.1186/s12884-023-05740-0
**Stoll**	2014	Canada	Midwifery Care in Rural and Remote British Columbia: Retrospective Cohort Study of Perinatal Outcomes of Rural Parturient Women With a Midwife Involved in Their Care, 2003 to 2008	MLCC	Personal continuity of care	10.1111/jmwh.12137
**Stoll**	2023	Canada	Perinatal outcomes of midwife-led care, stratified by medical risk: a retrospectivecohort study from British Columbia (2008–2018)	MLCC	Personal continuity of care	10.1503/cmaj.220453
**Styles**	2020	Australia	Implementation and upscaling of midwifery continuity of care: The experience of midwives and obstetricians	MLCC	Personal continuity of care	10.1016/j.wombi.2019.08.008
**Sutcliffe**	2012	Review	Comparing midwife-led and doctor-led maternity care: a systematic review of reviews	MLCC	Personal continuity of care	10.1111/j.1365-2648.2012.05998.x
**Symon**	2015	Scotland	Exploration of preterm birth rates associated with different models of antenatal midwifery care in Scotland: Unmatched retrospective cohort analysis	Midwife led antenatal care	Care by a midwife	10.1016/j.midw.2015.02.012

First author	Year	Country	Title	Element of Organisation	Group of elements of organisation	DOI
**Symon**	2019	Scotland	Using a quality care framework to evaluate user and provider experiences of maternity care: A comparative study	MLCC	Personal continuity of care	10.1016/j.midw.2019.03.001
**Tafe**	2023	Australia	Exploring women's experiences in a midwifery continuity of care model following a traumatic birth	Private care by midwife	Personal continuity of care	10.1016/j.wombi.2023.01.006
**Tait**	2024	Review	Nurse/midwife-to-patient ratios: A scoping review	Midwife led intrapartum care	Care by a midwife	10.1080/10376178.2024.2318361
**Talukdar**	2021	Review	A scoping review of evidence comparing models of maternity care in Australia	MLCC	Personal continuity of care	10.1016/j.midw.2021.102973
**Tenfielde**	2022	USA	Reducing Disparities in Postpartum Care Utilization: Development of a Clinical Risk Assessment Tool	Shared care (explicit shared interprofessional responsibility for the client)	Interventions to enhance inter-disciplinary collaboration	10.1111/jmwh.13461179
**Thiessen**	2016	Canada	Maternity Outcomes in Manitoba Women: A Comparison between Midwifery-led Care and Physician-led Care at Birth	Midwife led intrapartum care	Care by a midwife	10.1111/birt.12225
**Thomas**	2022	USA	Birth equity on the front lines: Impact of a community-based doula program in Brooklyn, NY	Continous support by a doula	Personal continuity of care	10.1111/birt.12701
**Thompson**	2023	USA	Postpartum care for parent-infant dyads: A community midwifery model	Midwife led post natal care	Care by a midwife	10.1111/birt.12822
**Thornton**	2016	USA	Cesarean Outcomes in US Birth Centers and Collaborating Hospitals: A Cohort Comparison	Birthcentre	Place of birth - outside the obstetric unit	10.1111/jmwh.12553

First author	Year	Country	Title	Element of Organisation	Group of elements of organisation	DOI
**Thornton**	2017	USA	Effect of Birth Center Care on Clinical and Cost Outcomes	Birthcentre	Place of birth - outside the obstetric unit	Thornton PD. Effect of Birth Center Care on Clinical and Cost Outcomes (Doctoral dissertation, University of Illinois Chicago).
**Tietjen**	2021	Germany	Model of care and chance of spontaneous vaginal birth: a prospective, multicenter matched-pair analysis from North Rhine-Westphalia	Birthcentre	Place of birth - outside the obstetric unit	10.1186/s12884-021-04323-1
**Tingstig**	2012	Sweden	Satisfaction with a Modified Form of In-Hospital Birth Center Care Compared with Standard Maternity Care	MLCC	Personal continuity of care	10.1111/j.1523-536X.2012.00533.x
**Toohill**	2019	Australia	Socioeconomic differences in access to care in Australia for women fearful of birth	MLCC	Personal continuity of care	10.1071/AH17271
**Toth**	2020	USA	Early impact of the implementation of Medicaid episode-based payment reforms in Arkansas	Bundled payment	Alternative payment model (other than fee for service)	10.1111/1475-6773.13296

First author	Year	Country	Title	Element of Organisation	Group of elements of organisation	DOI
**Turner**	2022	UK	The association between midwifery staffing levels and the experiences of mothers on postnatal wards: Cross sectional analysis of routine data	Midwife led post natal care	Care by a midwife	10.1016/j.wombi.2022.02.005
**van der Kooy**	2016	The Netherlands	Different settings of place of midwife-led birth: evaluation of a midwife-led birth centre	Birthcentre	Place of birth - outside the obstetric unit	10.1186/s40064-016-2306-2
**van der Kooy**	2017	The Netherlands	Planned home compared with planned hospital births: mode of delivery and Perinatal mortality rates, an observational study	Homebirth	Place of birth - outside the obstetric unit	10.1186/s12884-017-1348-y
**Van Montfort**	2020	The Netherlands	Impact on perinatal health and cost-effectiveness of risk-based care in obstetrics: a before-after study	Multidisciplinairy consultations and care pathways	Interventions to enhance inter-disciplinary collaboration	10.1016/j.ajog.2020.02.036
**van Wijngaarden**	2024	The Netherlands	Working in smaller teams in community midwifery practices to foster continuity of carer: Midwives' experiences – A qualitative study in the Netherlands	MLCC	Personal continuity of care	10.1016/j.wombi.2024.101663
**Vanderlaan**	2023	USA	Midwifery Workforce Density Moderates the Association Between Independent Practice and Pregnancy Outcomes	Desity of independent midwives in a state/region	Care by a midwife	10.1111/jmwh.13500
**Velthuijs**	2024	The Netherlands	Outcome of induction of labour at 41 weeks with foley catheter in midwifery-led care	Redistribution of care from medical specialist to community midwife	Care by a midwife	10.1016/j.midw.2024.104026

First author	Year	Country	Title	Element of Organisation	Group of elements of organisation	DOI
**Walker**	2023	England	OptiBreech collaborative care versus standard care for women with a breechpresenting fetus at term: A pilot parallel group randomised trial to evaluate the feasibility of arandomised trial nested within a cohort	Multidisciplinairy consultations and care pathways	Interventions to enhance inter-disciplinary collaboration	10.1371/journal.pone.0294139
**Wallace**	2024	USA	An alternative model of maternity care for low-risk birth: Maternal and neonatal outcomes utilizing the midwifery-based birth center model	Birthcentre	Place of birth - outside the obstetric unit	10.1111/1475-6773.14222
**Wassen**	2023	Review	Child and maternal benefits and risks of caseload midwifery - a systematic reviewand meta-analysis	MLCC	Personal continuity of care	10.1186/s12884-023-05967-x
**Watkins**	2021	Australia	Labouring Together: Women's experiences of "Getting the care that I want and need"in maternity care	Shared decision making	Woman centred care	10.1016/j.midw.2022.103420
**Wegrzynowska**	2023	Poland	Investment in the peace of mind? How private services change the landscape of maternity care in Poland	Private care by midwife	Personal continuity of care	10.1016/j.socscimed.2023.116283
**Wernham**	2016	New Zealand	A Comparison of Midwife-Led and Medical- Led Models of Care and Their Relationship to Adverse Fetal and Neonatal Outcomes: A Retrospective Cohort Study in New Zealand	MLCC	Personal continuity of care	10.1371/journal.pmed.1002134
**Whitburn**	2024	Australia	Women's views and experiences of a new Midwifery Group Practice model in rural Australia	MLCC	Personal continuity of care	10.1016/j.wombi.2024.101603
**Wiegerink**	2019	The Netherlands	Intrapartum and neonatal mortality in low-risk term women in midwife-led care and obstetrician-led care at the onset of labor: A national matched cohort study	Midwife led intrapartum care	Care by a midwife	10.1111/aogs.13767
**Wilkes**	2015	Australia	Reforming maternity services in Australia: Outcomes of a private practice midwifery service	MLCC	Personal continuity of care	10.1016/j.midw.2015.05.006

First author	Year	Country	Title	Element of Organisation	Group of elements of organisation	DOI
**Wong**	2015	Australia	Getting the first birth right: A retrospective study of outcomes for low-risk primiparous women receiving standard care versus midwifery model of care in the same tertiary hospital	MLCC	Personal continuity of care	10.1016/j.wombi.2015.06.005
**Yonemoto**	2013	Review	Schedules for home visits in the early postpartum period	Supportive care	Woman centred care	10.1002/14651858.CD009326.pub4
**Young**	2023	Australia	"Make it better for the women and babies who come after me": Findings from women in Australia completing the international Babies Born Better survey	Supportive care	Woman centred care	10.1111/birt.12762
