# Supplementary material for: Organising maternal and newborn care in high-income countries: a scoping review of organisational elements and their association with outcomes
Source: BMJ Open. 2025 Dec 14;15(12):e107624. doi: 10.1136/bmjopen-2025-107624 (PMC12706211; doi:10.1136/bmjopen-2025-107624)
Supplement: Supplementary file 4 [file bmjopen-2025-107624.reviewer_comments.pdf]

PEER REVIEW HISTORY

BMJ Open publishes all reviews undertaken for accepted manuscripts. Reviewers are asked to complete a checklist review form and are provided with free text boxes to elaborate on their assessment. These free text comments are reproduced below.

ARTICLE DETAILS

Title (Provisional)

ORGANISING MATERNAL AND NEWBORN CARE IN HIGH-INCOME COUNTRIES: A SCOPING REVIEW OF ORGANISATIONAL ELEMENTS AND THEIR ASSOCIATION WITH OUTCOMES

Authors

Liebregts, Jolanda; Goodarzi , Bahareh; Valentijn, Pim; Downe, Soo; Erwich, Jan Jaap; Burchell, George; Batenburg, Ronald; de Jonge, Ank; Verhoeven, Corine J. M.; Study Group, VOICE

VERSION 1 - REVIEW

**Reviewer**	**1**
**Name**	**Stierman, Elizabeth**
**Affiliation**	**Johns Hopkins University Bloomberg School of Public Health, International Health**
**Date**	**12-Sep-2025**
**COI**	**None**

Thank you for sharing your article. It is an ambitious project, and the authors have reviewed and synthesized an extensive breadth of literature on this topic. This is commendable.

However, I am unable to recommend publication of the article in its current form, without a major revision. My primary concern is that the conclusions go further than what can be justified within the methodological limitations of a scoping review. A scoping review aims to systematically map the breadth of evidence available on a given topic in the published literature. The author appropriately state this as their objective (i.e., “explored the extent and type of evidence on…” in last paragraph of Introduction), but the authors do not only report the extent and type of evidence published on the topic. They also report on the impact of the interventions (e.g., “high-certainty evidence from RCTs and systematic reviews indicated that midwife-led continuity of care was associated with….” In Table 2; “Personal continuity of care was associated with better health outcomes…” in the Results narrative). Such statements need to be justified by careful review of evidence and considerations of the methodological quality of the primary studies and consideration of risk of bias; in this

example, it is unclear how the authors graded the evidence to determine it was high certainty. This level of assertion would be more appropriate for a systematic review of interventions, which includes an assessment of the risks of bias. A scoping review is not appropriate for evaluating the impact of intervention(s) on outcomes. Instead, I suggest the authors limit their results and discussion to what is possibly to evaluate within a systematic review, namely the "extent and type of evidence."). For example, the Results presented in Table 3 which map what evidence has been published on different dimensions of integrated care seems more appropriate for the methods of a scoping review.

In addition to this overarching concern, additional clarity is needed on the Methods authors employed, including:

1. Was the protocol prospectively registered? Were there any deviations from the original protocol?

2. The narrative description of the search strategy used with phrase "combined with", which suggests use of "AND" as a Boolean operator. I believe the authors used "OR" between certain concepts.

3. It is unclear how the two reviewers interacted with the ASReview for the screening of titles and abstracts. Did the reviewers validate the results from ASReview? Did they initially screen title and abstracts and feed this to the ASReview as part of its learning process? Please elaborate.

4. The authors state that "in the second stage, full-text screening was conducted by two reviewers." Was this done in duplicate?

5. The authors describe how they categorized the dimensions of integrated care using a deductive approach informed by the RMIC. However, it is unclear how they categorized "elements of organization" such as "personal continuity of care", "interventions to foster interdisciplinary collaboration", "care by a midwife" etc. Were these categories produced through an inductive or deductive approach? If deductive, what theory or framework were these based on?

**Reviewer**	**2**
**Name**	**Bayes, Sara**
**Affiliation**	**Edith Cowan Univ, School of Nursing and Midwifery**
**Date**	**19-Sep-2025**
**COI**	**None**

Thank you for the opportunity to review this manuscript, in which the authors share their scoping review of literature on organisational elements of maternal-newborn care and their

association with MNC outcomes. Please find my remarks below, including two suggestions for very minor revisions.

The problem is framed clearly and justifies the study. The methods are described in sufficient detail and are faithful to the chosen (JBI) methodology. The extracted data are organised effectively in accordance with pre-selected (RMIC) groupings, and the story they represent is conveyed efficiently and articulately.

The themes are thoughtfully considered and contextualised against relevant recent literature in the Discussion, and it is particularly good to see the consideration of implementation considerations in the second last paragraph. In my view, the final paragraph of the Discussion belongs in the Conclusions section, together with the authors' other recommendations.

The manuscript does require a careful read-through for grammar, punctuation (e.g., in the first sentence in the 'Collaborators' section, there should be another comma after '...organisation of MNC').

Otherwise, I have no other suggestions for the authors and commend them on a thorough and valuable piece of work.

VERSION 1 - AUTHOR RESPONSE

**Reviewer: 1**	
Overarching concern: 'Instead, I suggest the authors limit their results and discussion to what is possibly to evaluate within a systematic review, namely the "extent and type of evidence."'	*We acknowledge the reviewer's concern and agree that the results and discussion should be limited to describing the extent and type of available evidence. As our study follows the JBI methodology for scoping reviews, we revised the manuscript to ensure that all findings are presented descriptively and in accordance with JBI guidance. Wording that could imply causal relationships or findings derived from this review was adjusted to clarify that associations were reported in the included publications. These revisions were made in Table 2, and throughout the Abstract, Results, Discussion, and Conclusion sections. Given the large number of (minor) textual edits, all changes are shown in track changes but are not listed individually here.*
Was the protocol prospectively registered? Were there any deviations from the original protocol?	*The protocol for this scoping review was prospectively published prior to conducting the review, and no deviations were made from the original protocol. The only modification was an updated literature search, which was performed because more than one year had passed between the initial search and the preparation of the manuscript. This clarification has been added to the Methods section. (P4, L28-31).*

The narrative description of the search strategy used with phrase "combined with", which suggests use of "AND" as a Boolean operator. I believe the authors used "OR" between certain concepts.	*The wording in the Methods section was adjusted to specify that the search terms were combined using the Boolean operator OR between related concepts (P5, L2-3).*
It is unclear how the two reviewers interacted with the ASReview for the screening of titles and abstracts. Did the reviewers validate the results from ASReview? Did they initially screen title and abstracts and feed this to the ASReview as part of its learning process? Please elaborate.	*In the Methods section, we now explain that both reviewers trained the ASReview model by labelling an initial set of records and key articles, after which screening proceeded in the prioritised order suggested by the model. Reviewers' ongoing decisions were fed back iteratively, and all inclusion/exclusion decisions were made and cross-checked by the reviewers. (P5, L24-34)*
The authors state that "in the second stage, full-text screening was conducted by two reviewers." Was this done in duplicate?	*The Methods section was revised to clarify the full-text screening procedure. We specified that screening was initially conducted in duplicate through a staged calibration process in which both reviewers independently assessed three consecutive sets of 25 full-texts. Disagreements were resolved through discussion or, when necessary, consultation with a third reviewer. As agreement was reached in the third set, one reviewer completed the remaining full-text screening while continuing to discuss any uncertainties with the other reviewers. (P6, L3-8)*
The authors describe how they categorized the dimensions of integrated care using a deductive approach informed by the RMIC. However, it is unclear how they categorized "elements of organization" such as "personal continuity of care", "interventions to foster interdisciplinary collaboration", "care by a midwife" etc. Were these categories produced through an inductive or deductive approach? If deductive, what theory or framework were these based on?	*The Methods section was expanded to clarify how the organisational elements were categorised. We now specify that an inductive approach was first used to identify specific organisational elements from the included studies, followed by a deductive mapping of these elements onto the dimensions of the Rainbow Model of Integrated Care (RMIC). The description of this combined approach and the role of the RMIC determinants has been added to the Methods section and illustrated in Table 3. Disagreements between reviewers were resolved through discussion or consultation with a fourth reviewer. (P6, L12-18)*
**Reviewer: 2**	
In my view, the final paragraph of the Discussion belongs in the Conclusions section, together with the authors' other recommendations.	*We agree with the reviewer that the final paragraph of the Discussion contained content that fits better in the Conclusions section. We therefore integrated into the revised Conclusions, ensuring a clearer distinction between discussion of findings and concluding implications.*
The manuscript does require a careful read-through for grammar, punctuation (e.g., in the first sentence in the 'Collaborators' section, there should be another comma after '...organisation of MNC').	*The entire manuscript was carefully proofread, and grammar and punctuation were adjusted throughout.*
